# Insights on the interaction of SARS-CoV-2 variant B.1.617.2 with antibody CR3022 and analysis of antibody resistance

**DOI:** 10.1186/s43141-023-00492-y

**Published:** 2023-03-20

**Authors:** Sandhya KS, Achuthsankar S. Nair

**Affiliations:** 1 Department of Computational Biology and Bioinformatics, University of Kerala, Kerala Thiruvananthapuram, India; 2 Malankara Catholic College, Mariagiri, Kaliakkavilai, Kanyakumari, 629153 Tamil Nadu India

**Keywords:** Homology modelling, SARS-CoV-2, Delta, CR3022, Molecular dynamics, Molecular docking

## Abstract

**Background:**

The existence of mutated *Delta* (B.1.617.2) variants of SARS-CoV-2 causes rapid transmissibility, increase in virulence, and decrease in the effectiveness of public health. Majority of mutations are seen in the surface spike, and they are considered as antigenicity and immunogenicity of the virus. Hence, finding suitable cross antibody or natural antibody and understanding its biomolecular recognition for neutralizing surface spike are crucial for developing many clinically approved COVID-19 vaccines. Here, we aim to design SARS-CoV-2 variant and hence, to understand its mechanism, binding affinity and neutralization potential with several antibodies.

**Results:**

In this study, we modelled six feasible spike protein (S1) configurations for Delta SARS-CoV-2 (B.1.617.2) and identified the best structure to interact with human antibodies. Initially, the impact of mutations at the receptor-binding domain (RBD) of B.1.617.2 was tested, and it is found that all mutations increase the stability of proteins (*ΔΔ*G) and decrease the entropies. An exceptional case is noted for the mutation of G614D variant for which the vibration entropy change is found to be within the range of 0.133–0.004 kcal/mol/K. Temperature-dependent free energy change values (*Δ*G) for wild type is found to be − 0.1 kcal/mol, whereas all other cases exhibit values within the range of − 5.1 to − 5.5 kcal/mol. Mutation on spike increases the interaction with the glycoprotein antibody CR3022 and the binding affinity (CLUSpro energy =  − 99.7 kcal/mol). The docked Delta variant with the following antibodies, etesevimab, bebtelovimab, BD-368–2, imdevimab, bamlanivimab, and casirivimab, exhibit a substantially decreased docking score (− 61.7 to − 112.0 kcal/mol) and the disappearance of several hydrogen bond interactions.

**Conclusion:**

Characterization of antibody resistance for Delta variant with respect to the wild type gives understanding regarding why Delta variant endures the resistance boosted through several trademark vaccines. Several interactions with CR3022 have appeared compared to Wild for Delta variant, and hence, it is suggested that modification on the CR3022 antibody could further improve for the prevention of viral spread. Antibody resistance decreased significantly due to numerous hydrogen bond interactions which clearly indicate that these marketed/launched vaccines (etesevimab) will be effective for Delta variants.

**Graphical Abstract:**

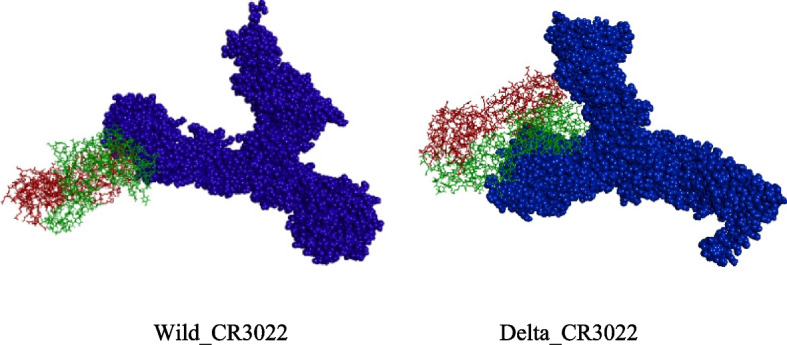

**Supplementary Information:**

The online version contains supplementary material available at 10.1186/s43141-023-00492-y.

## Background

The “human angiotensin-converting enzyme 2” (hACE2) is a potential receptor for the deadly virus SARS-CoV-2. This interaction causes the life-threatening infection “COVID-19,” and so far, there is no optimized medicine with a suitability of 100% for treating this disease. From 3rd January 2020 to 23rd December 2022, there have been 651,918,402 confirmed cases of COVID-19 with 6,656,601 deaths [1]. As per records, the first case of COVID-19 in India was reported on 30 January 2020, and a rapid escalation of cases was noted in mid-March 2020 [2]. Coronaviruses belong to family of Coronaviridae and the subfamily of Coronavirinae. The four genera of Alphacoronavirus, Betacoronavirus, Gammacoronavirus, and Deltacoronavirus were identified in the subfamily [3]. Like the SARS-related coronavirus implication in 2003 SARS outbreak, SARS-CoV-2 has been classified within the Coronaviridae family, Betacoronavirus genus, and Sarbecovirus subgenus [4, 5]. In general, coronaviruses undergo frequent recombination and the mechanism of recombination and copy-choice replication in which gene material switches from one RNA template molecule to another [3, 6]. Hence, SARS-CoV-2 continuously evolves in terms of genetic code or mutations that occur during the replication of the genome [7].

After the first phase of COVID-19 pandemic, the world realized that new variants are emerging with alarmingly high ability for human-to-human transmission. The mutated variants of SARS-CoV-2 are commonly classified into two categories, such as “variants of concern” and “variants of interest.” Based on the Pango lineage nomenclature [8], variants of concern (VIO) are grouped into four more categories, namely B.1.1.7, B.1.351, P.1, and B.1.617.2. A recent study indicated that a few VIOs are, for example, B.1.1.7 observed in the UK, and B.1.351 observed in South Africa are quite obstinate to monoclonal antibodies against N-terminal domain (NTD) or receptor-binding domain (RBD) [9]. The K484K substitution in the immunodominant epitope of B1.1.7 and B.1.351 variants could be the reason to resist the neutralization of antibodies [9, 10]. This implies that the various mutations in the NTD and RBD of spike protein can have high affinity towards the ACE2 receptor. Gamma variant (P.1) shows significant resistance to multiple monoclonal antibodies, convalescent plasma, and vaccine sera [11]. Similar to B.1.351, K484K mutation is also seen in P.1, but NTD mutation may be the cause of neutralization of antibodies. At the end of May 2021, a new variant emerged in India named B.1.617.2 (δ) which exhibits the mutation of L452R and T478K in RBD with a total of 12 mutations in spike protein compared to wild type [12]. Interestingly, this lineage lacks mutation of amino acid residues 501 and 484. Moreover, the efficacy of the anti-NTD and anti-RBD mAbs antibodies is also very weak.

The trimeric spike protein consists of two distinct parts (S1 and S2) with a range of 1 to 1273 amino acid residues. The surface protruded part, S1, is RBD which engages with the host cell receptor ACE2 of the human body, while S2 consists of hydrophobic fusion peptide and two heptad repeat regions. The S2 part is utilized for the fusion and entry of the virus with the assistance of cell surface serine protease TMPRSS2. The viral transmission can be blocked by antibodies or drugs. However, no antibodies are 100% effective, though the development of vaccine designs was widely addressed across the world. The change of the structure by mutation as the virus passes from person to person makes it difficult to control. Tracking the latest updates on spike protein in UniProtkb-P0TDC2 clearly indicates that the first mutation of SARS-CoV-2 was detected in late January 2020 (PDB ID: 6XS6) on the spike protein. This is superseded by glycine with aspartic acid (D617G) and circulating this single mutation in the reported lineage of spike protein all over the world [13]. This mutation does not reduce the affinity with ACE2 but mediates the transduction of cells. Other single mutations on the spike protein are Q493N, Q493Y, and N501T. The B.1.617.2 lineage is a subset of B.1.617 which emerged in October 2020, and the other two subsets are B.1.617.1 and B.1.617.3. It is observed that L452R mutation on spike protein persists in three subsets and B.1.617 lineage [14, 15]. Apart from that P681R, mutation can be seen for B.1.617.2 [16]. On the other hand, E484Q mutation is absent in B.1.617.2 lineage but present in all other cases. Moreover, deletion of sequences 157 and 158 was seen only in B.1.617.2. Major mutations are present in NTD and RBD of the S1 domain.

In this study, we focus on the S1 spike protein of B.1.617.2 lineage which makes a viral transmission more than other variants. All mutations are characterized and identified in India and compared with the reference sequence of wild type (accession code: YP_009724390). Recently, this variation has increased in proportion and become dominant over other subsets of lineage in India. We demonstrate six homology modelling and characterization of spike protein from a sequence of B.1.617.2 which contains various mutations. To the best of our knowledge, spike protein having full length (1273 amino acids) of Delta with CR3022 has not been studied until now. Moreover, it is necessary to understand the influence of all amino acids (1–332 and 527 to 1273 amino acids) along with mutated amino acids in the RBD (333 to 527 amino acids). Furthermore, a deep insight into the dynamics of full-length mutated spike protein with antibodies is analyzed in terms of stability. Owing to the various mutations on spike protein, their interaction with human antibody CR3022 is also scrutinized and compared with the wild spike-antibody complex at the molecular level.

## Methods

### Sequence retrieval, alignment, secondary structure, and homology modelling

All the sequences of B.1.617.2 lineage were downloaded from the National Center for Biotechnology Information (NCBI) virus SARS-CoV-2 data hub. QWA32943.1, QVY49647.1, QWA33015.1, QWC92735.1, QVY49671.1, QWE51975.1, QWE51929.1, QWK39339.1, QWK39327.1, QWC36279.1, and QWK39303.1 are sequence accession codes. It is also observed that all sequence accession codes are complete for RBD which contains 1 to 1273 sequences. D142G, L154E, L382V, R452L, Q484E, R681P, I95T, and G614D mutations are considered in these sequence analyses. Online clustal omega software and NCBI site were used for the alignment. Sequence homology modelling is done with the use of SWISS-MODEL with the given template [17]. All updates of spike protein were found in the P0DTC2 of UniProt knowledge base (UniProtKB) repository. From that, 6VYB was selected for the target template. SWISS-MODEL server homology modelling pipeline computes models by extracting initial sequence and template using ProMod3. OpenMM library is used for the computations, and CHARMM22/CMAP force field was utilized for parameterization. Best models are estimated by Global Model Quality Estimation (GMQE), quality model energy analysis 4 (QMEAN4) score, and Ramachandran plot obtained from SWISS built model. GMQE estimates the properties from target template alignment and reference template structure. The score value near to 1 reflects the accuracy and reliability of each model. QMEAN4 represents the degree of nativeness of the structural features seen in the model. QMEAN score around zero indicates the good agreement between the template and the built model, while below 4 indicates low quality. The secondary structure was predicted using the PSI-blast-based secondary structure (PSIPRED) online server [18]. The server predicts helix, strand (sheet), and coil from the given input sequences. The structure determination is based on the position-specific scoring matrices generated by PSI-BLAST.

### Protein dynamics and molecular docking

To assess the impact of various mutations in the modelled structures, we used the DynaMut server [19]. Wild type is uploaded along with given corresponding mutations into the server. The server utilizes the vibrational entropy changes for finding dynamicity and stability. Each mutation is analyzed by DynaMut online server. For comparison, we also obtained DUET, mCSM, and SDM values from the DynaMut server. The free energy change between Wild type and its mutated form (ΔΔG) values are expressed in kcal/mol for each mutation in the model. Proteins stabilize/destabilize based on the positive/negative values of *ΔΔ*G. Server output is based on the “multiple mutation” list option. SCooP server for all models and the wild type were utilized for predicting the Gibbs–Helmholtz free energy due to folding transition [20]. Furthermore, the change in enthalpy (*ΔH*_m_) and heat capacity upon folding (ΔC_P_), the melting temperature (*T*_m_), and the folding free energy at room temperature (ΔG) were also obtained from the same server tool.

Molecular docking between the spike protein model and the antibody CR3022 was performed using the CLUSpro2.0 server [21]. Antibody mode was set, and attractor residues of both antigen and antibody were given based on the knowledge information from pdb of 6W41. Residues except attractors are non-CDR regions, which are uploaded in the form of pdb. The crystal structure of CR3022 (PDB id 7BWJ) was downloaded from the RSCPDB database. The unmutated spike protein was taken from PDB id 6VYB which was determined by cryo-electron microscope with a resolution of 3.4 Å. Proteins are visualized using VMD and PyMOL. All proteins are free from water, ligands, and ions. CLUSpro is based on the fast Fourier correlation approach implemented in the PIPER. In each complex, spike protein is subjected to 70,000 rotations, and out of the 70,000 rotations, 1000 rotations/translation combinations having lowest score were selected. Subsequently, a greedy clustering of these spike positions and their neighboring position within 9-Å c-alpha RMSD radius was performed. This is known as a cluster center, and neighboring positions are the members of this cluster center. These were then removed from the cluster center to acquire a new cluster center. In each 70,000 rotation, sampling of 10^9^ positions of ligand relative to the receptor was performed. From that sampling, 1000 positions were selected as the top score with lowest energy structures. Such positions are used to find the largest clusters that will use the most likely models of the complex. Six models and wild type were docked with antibody CR3022 using CLUSpro server. The server provides total energy which is the sum of 0.50 *E*_rep_, 0.20 *E*_att_, 600 *E*_elec_, and 0.25 *E*_DARS_, where *E*_rep_ and *E*_att_, respectively, denote repulsive van der Waals energy and attractive van der Waals energy values; *E*_DARS_ represents pairwise structure-based potential, and the term *E*_elec_ represents electrostatic energy. Binding of wild type with CR3022 yields 27 cluster protein-antibody complexes. In the docking analysis, we considered interaction in the residue between 321 and 521, and all interactions are within a cutoff range of 2.7 Å.

Molecular dynamics simulations were carried out for the complex and uncomplex structures using GROMACS 2021 [22]. The best-docked complex structure is used for the simulation studies. The GROMOS96 43al force field was used for proteins, antibodies, water, and ions. SPCE was selected as a solvent model with cubic box. The system was neutralized by adding 7 chlorine ions based on the total charges. A total number of water molecules is 452,221. Long-range electrostatic interactions were calculated using particle mesh Ewald method with a cutoff value of van der Waals interactions, 1 nm. LINCS algorithm was utilized for holonomic constraints. Temperature and pressure were maintained using V-rescale of modified Berendsen thermostat and Parrinello-Rahman method in NPT. The protein was relaxed using energy minimization for 50,000 steps. In the next step, the protein should equilibrate with the solvent and ions; hence, it should pass through two phases. The first one is a canonical ensemble (NVT) for 100 ps. For the second phase, we stabilized the pressure and density of systems using NPT ensemble for 100 ps. For both phases, we used a leap-frog integrator. After achieving constant temperature, pressure, and density, we have conducted production molecular dynamics for 100 ns. Root-mean-square deviation (RMSD), root-mean-square fluctuation (RMSF), solvent accessible surface area (SASA), radius of gyration (Rg), and hydrogen bond between protein and antibodies were calculated from molecular dynamics trajectory files.

### MM/GBSA calculations

MM/GBSA is used for finding binding energy and decomposition free energy contributions to the binding energy of protein-antibody complex. HawkDock server employs MM/GBSA based on ff02 force field with iGBOBC1 model and implicit solvent model [23]. The given systems were minimized by 5000 steps. Van der Waals cutoff distance of 12 Å with 2000 cycles is for steepest descent and 30,000 cycles for conjugate gradient minimization. The last frame of the trajectory files was converted into pdb files for MMGBSA calculation.

## Results and discussion

### Sequences and mutations

**Fig. 1:**
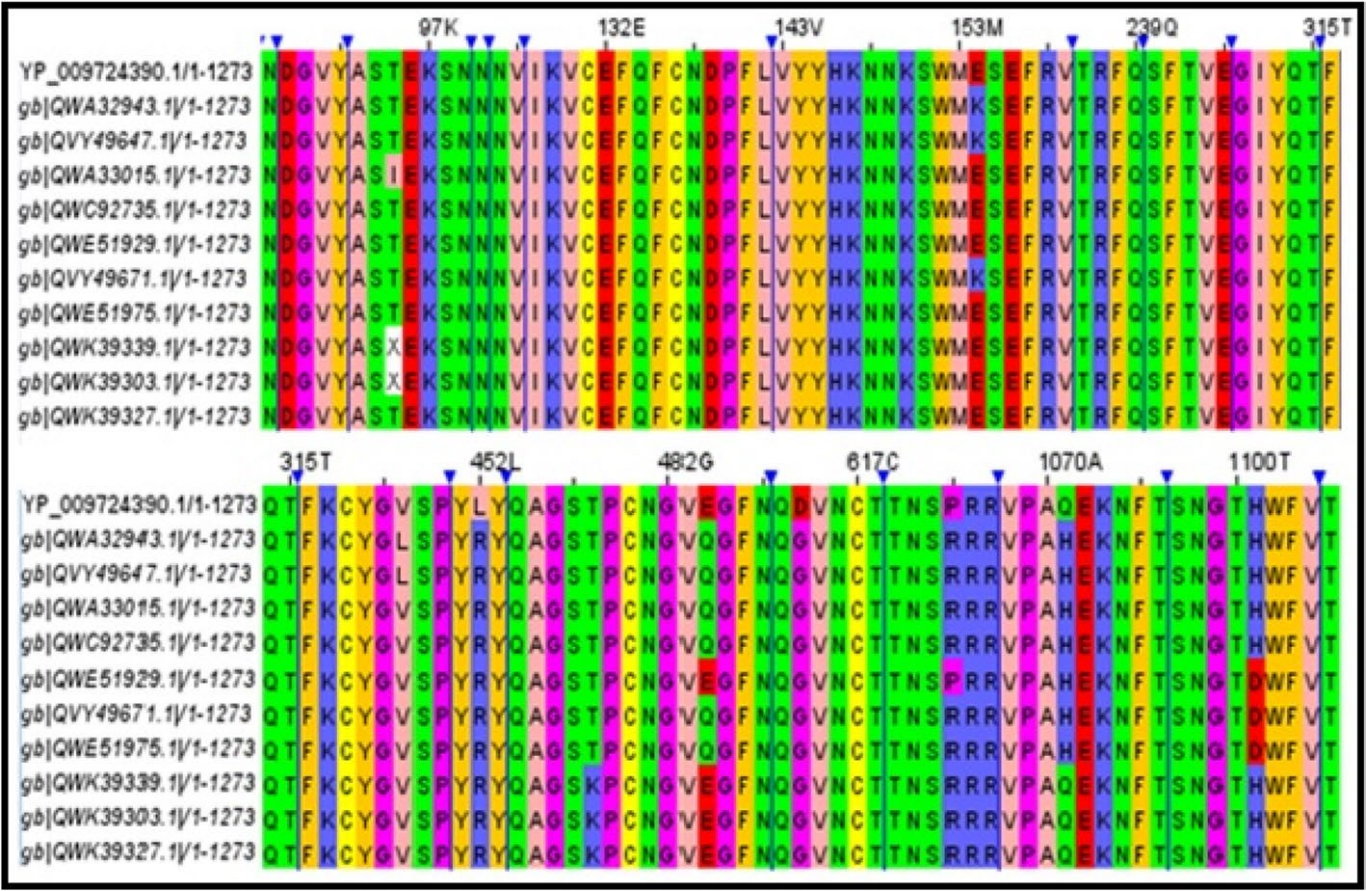
Eleven sequences (including reference sequence) between 1 and 1273 (regions having no changes in the sequence are hidden, and mutated sequences are shown in color variation in the alignment)

Figure 1 demonstrates eleven sequences obtained from the NCBI virus data hub. In this figure, the first sequence (YP_009724390.1/1–1273) corresponds to wild type, whereas the proceeding sequences correspond to mutated forms of B.1.617.2. The collected sequences contain mutations at the position numbers 19, 95, 142, 154, 382, 452, 478, 484, 614, 681, 950, 1070, 1100, and 1150. In the sequence IDs QVY49647.1 and QWA32943.1, mutations were noticed at three common positions V382L, R452L, and Q484E (triple mutations). On the other hand, QVY49647.1 shows one additional mutation at R681P. The sequence IDs QWC92735.1, QVY49671.1, and QWA33015.1 exhibit R452L and Q484E double mutations, whereas the sequence QWE51929.1 show a single mutation (R452L) in the RBD of S1. For better clarity, the complete mutations of each model are summarized in Table 1. One can notice in the table that the mutation at D614G is common in all the cases. The sequences QWC92735.1, QVY49671.1, and QWA33015.1 show many mutations below and above the receptor-binding domain (RBD) of the spike S1. It is well known that RBD is crucial for viral interaction with ACE2; nonetheless, we have taken complete sequences of spike (1 to 1273). The sequence QWE51929.1 shows two mutations at G142D and G1100D. It is expected that many mutations other than in RBD can change the spike protein structural feature for influencing human antibody CR3022. This can be strong or weak; hence, such inclusion of mutations is important in order to understand the exact interaction of spike with CR3022.

**Table 1:** List of complete mutations for each model used for the present study

Models	Complete mutations in each model
Model 1	D142G, K154V, L382V, R452L, Q484E, R681P, G614D
Model 2	R452L, Q484E, R681P, G614D
Model 3	I95T, D142G, R452L, Q484E, G614D
Model 4	D142G, K154V, R452L, Q484E, G614D
Model 5	D142G, K154V, L382V, R452L, Q484E, D142G, G614D
Model 6	D142G, R452L, G614D

### Characterization of models

**Fig. 2:**
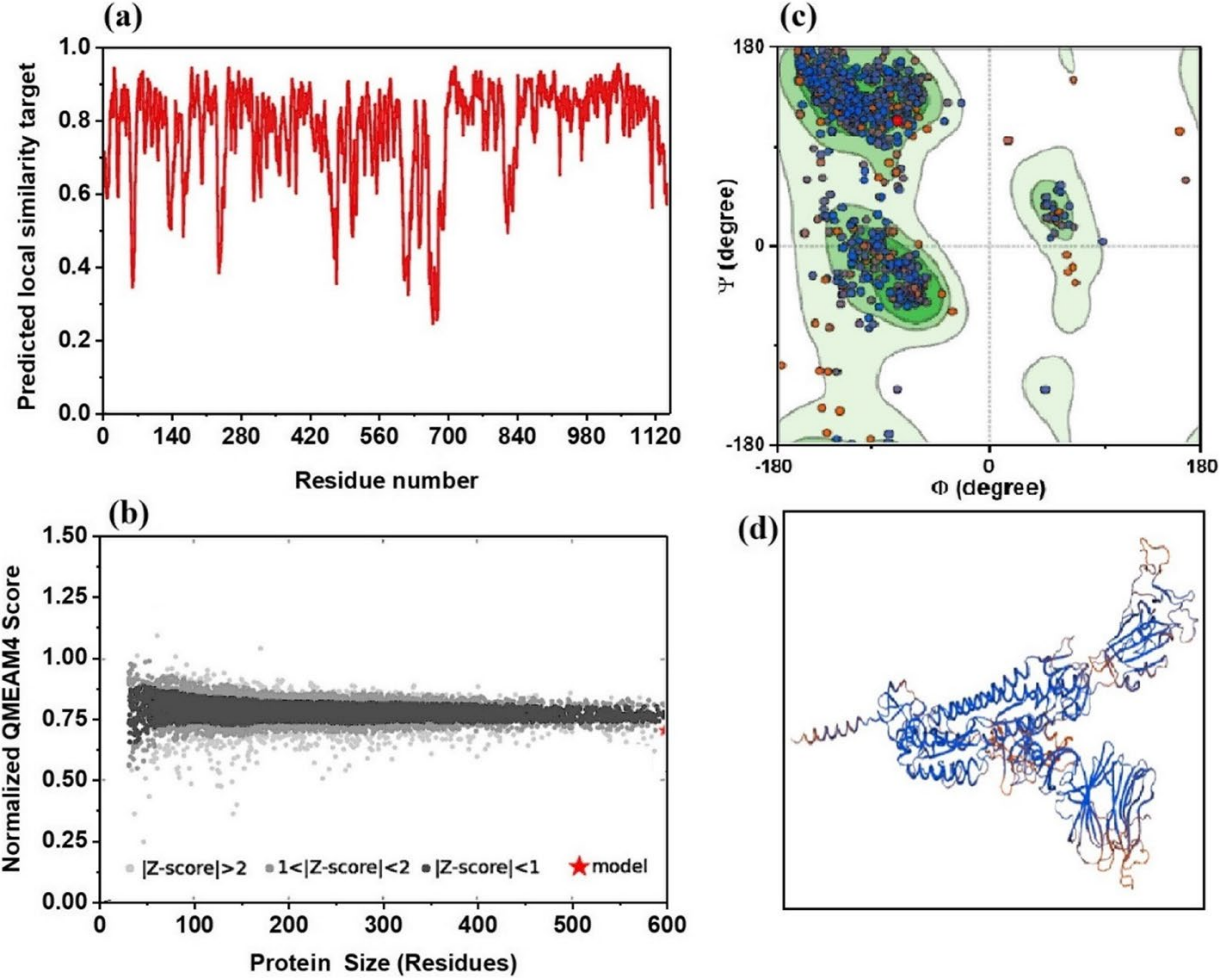
**a** Profiles obtained for the local similarity to template versus residue number, **b** protein size vs QMEAN4 score, **c** Ramachandran plot of QWA32943 sequence, and **d** homology Model 1

Initially, the local quality was evaluated (SWISS-MODEL) by means of the predicted local similarity to template with the residue number. As seen in Fig. 2a, majority of residues of models are well above the cutoff score (0.60). The target template alignment indicates the highest quality-built model of sequence identity for all models above 99%. It was further analyzed by GMQE for the expected quality of the model. For good quality, measurement between the submitted template and sequence should lie in between 0 and 1. All models show near to 1 (lies between 0.66 and 0.64) indicating the reliability of the quality of the model estimation. Comparison with a non-reductant set of PDB structures is obtained by plotting QMEAN4 score with respect to the size of the residue (Fig. 2b). The observed score distribution suggests that the obtained model reflects a native-like structure. Ramachandran plot was used to show how well two dihedral angles (Ψ and Φ) of amino acids of proteins are seen in the allowed or favorable regions and hence to assess the quality of the protein model even in the absence of experimental data. This gives an idea regarding which combination of angles is possible. Furthermore, dihedral angles of amino acid residues determine the geometry of its attachments and conformations of residues. Hence, many confirmations are not possible due to steric hindrance. The Ramachandran plot and the representative model structure for QWA32943 are provided in Fig. 2c and d. For the remaining cases (Models 2 to 5), the residue number vs local similarity, size vs QMEAN4 score, Ramachandran plot, and the corresponding model structure are given in the supporting information, Fig. S1.

Next, we carried out secondary structures prediction of all models, and they are shown in the SI (Fig. S3). All models show a change in the secondary structure due to the mutations. For instance, Model2 exhibit a change of coil to sheet due to E484Q, whereas L452R and P681G do not make any difference, but some neighboring residues are changed from helix to coil and sheets to coil. Interestingly, mutation at P681R as well as Q1071H does not make any change in the secondary structure of that particular site. It can be suggested that mutations inside RBD could make marginal changes in the secondary structure of the given protein.

### Stability of proteins

**Fig. 3:**
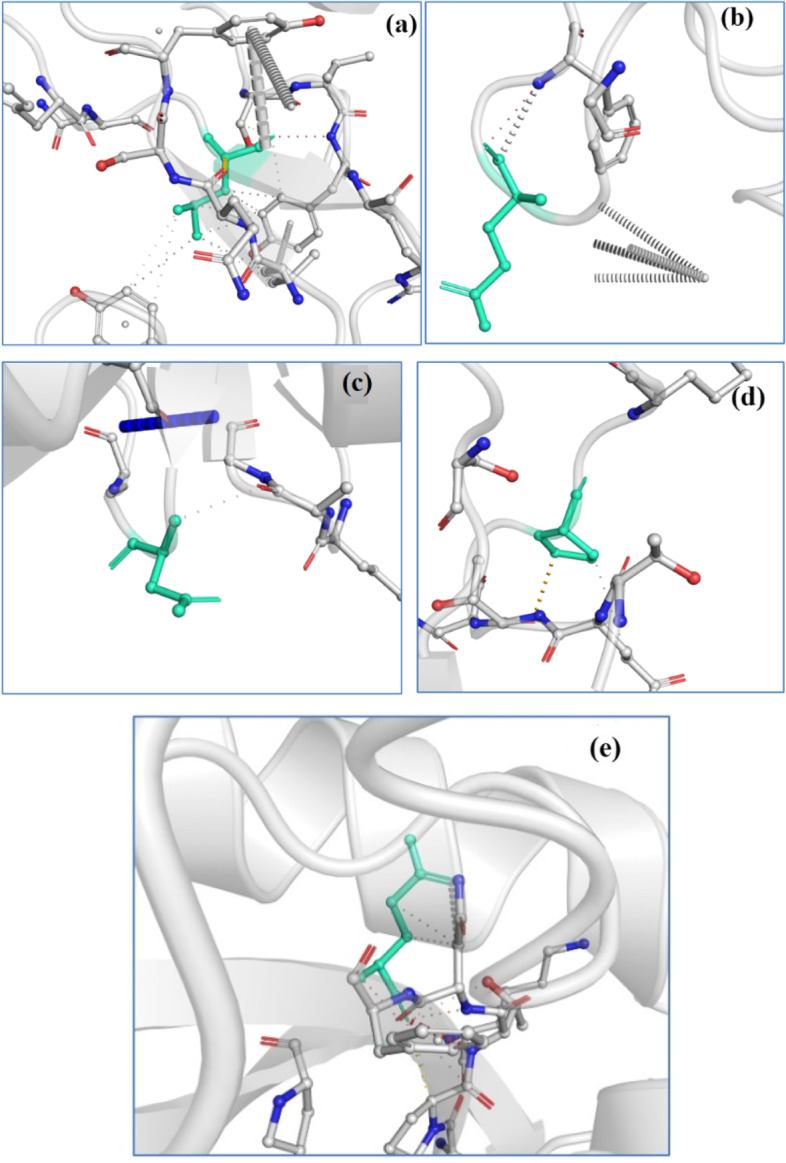
Contact of mutated amino acid residues of spike protein with CR3022. **a** L452R, **b** E484Q, **c** D614G, **d** P681R, and **e** Q1071H. The dots represent various weak interactions

In general, all mutations cause stabilization on the specific site [24], and it changes the protein conformational equilibria as well as the system dynamics. Therefore, it is necessary to understand the consequences of mutation on the molecular structure. In the literature, a valuable tool DynaMut is proposed for calculating the change in free energy folding between wild-type and mutated protein [25]. In the current study, we have estimated *ΔΔ*G through DynaMut for all the six models and observed that *ΔΔ*G lies between 0.138 and 1.689 kcal/mol. Table 2 gives us information of DynaMut results of Model 2, and their corresponding interacting residues are shown in Fig. 3. In all the cases, mutation on the site 614 exhibits high dynamic flexibility, which leads to entropy increase by 0.133 kcal/mol/K. Loss of a hydrogen between loop and α-helix could be the reason for the flexibility [26]. Change in vibration entropies (*ΔΔ*S) is found to be in between 0.133 and − 0.289 kcal/mol/K. The entropy changes cause the molecular flexibility which enhances the unfolding for the attachment of CR3022. Except for 614, all other mutations show decrease in the molecular flexibility. We observed that the trend observed for *ΔΔ*G through DynaMut is similar to that estimated through EnCoM except the G614D mutation. For comparison with DynaMut, we also used mCSM, SDM, and DUET, and the values are provided in the SI (Tables S1–S11). The data we summarized in the SI contains information regarding *ΔΔ*G and *ΔΔ*S for each mutation for all six models.

**Table 2:** *ΔΔ*G (kcal/mol) and *ΔΔ*S ENCoM and *ΔΔ*G DynaMut of Model 2

Serial no	AA from	AA to	Position	*ΔΔ*G ENCoM (kcal/mol)	*ΔΔ*S ENCoM (kcal/mol)	ΔΔG DynaMut (kcal/mol)
1	L	R	452	0.142	− 0.178	0.511
2	Q	H	1071	0.003	− 0.004	0.138
3	E	Q	484	0.07	− 0.088	0.439
4	D	G	614	− 0.106	0.133	0.548
5	P	R	681	0.373	− 0.467	0.467

We have also compared temperature-dependent stability curves using SCooP server for all models and the wild type (Fig. S2). The wild type shows the folding free energy change (ΔG) of − 0.1 kcal/mol at room temperature, whereas all other models exhibit *Δ*G between − 5.1 and − 5.5 kcal/mol. It means that various substitutions significantly affect the thermal stability of protein [27]. Furthermore, the wild type and all other models do not show significant difference between the melting point temperatures. Similarly, heat capacity upon folding also found to be in marginal difference. However, it is notable that the change in enthalpy for all models is less than − 100 kcal/mol, while a significant difference for *∆H*_m_ and *∆C*_p_ occurs for the Wild (− 2.6 kcal/mol and − 0.06 kcal/mol K) (Table S12).

### Docking analysis of spike protein and antibody CR3022

**Fig. 4:**
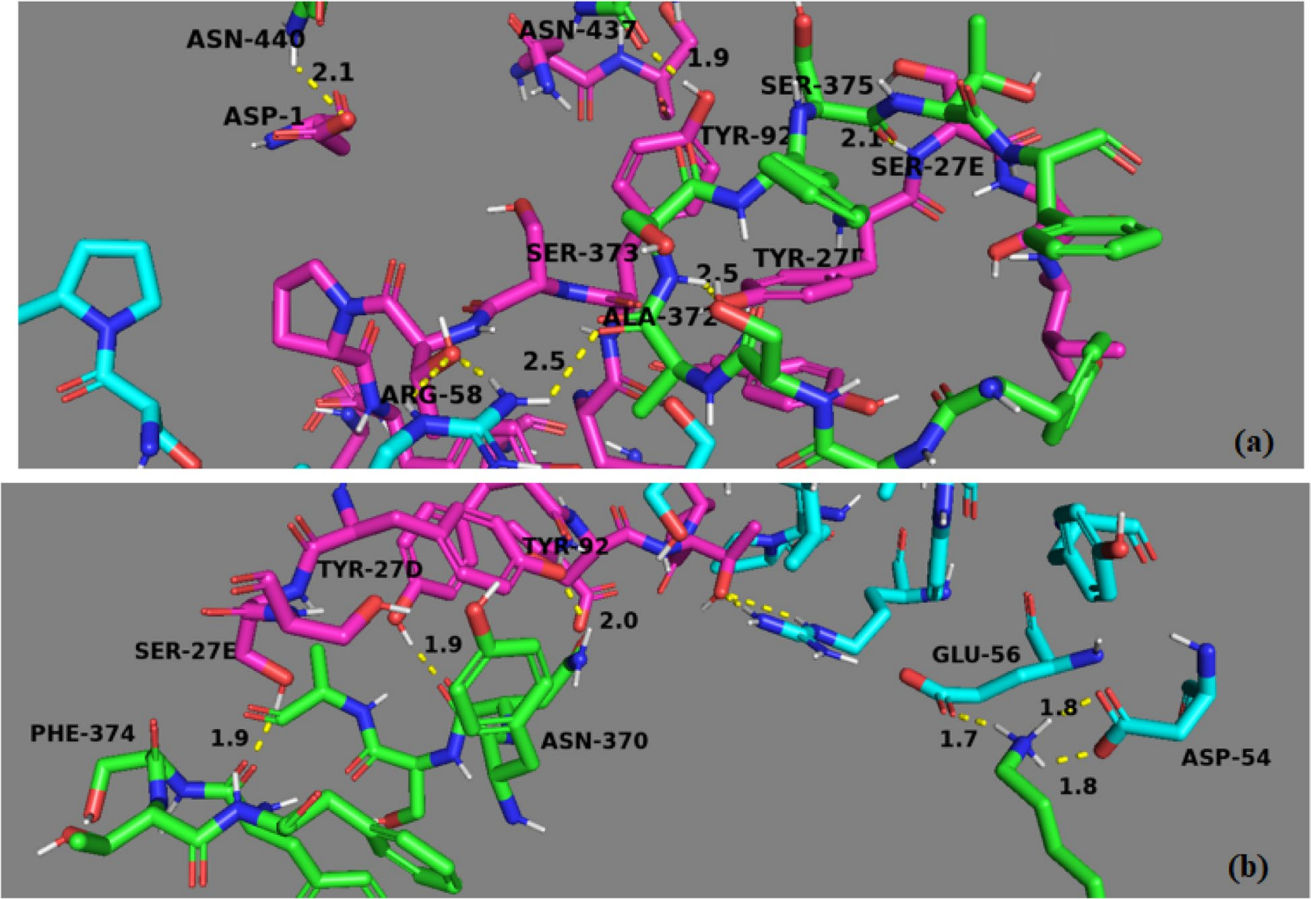
Docked systems of **a** Wild_CR3022 and **b** Model2_CR3022

Enzyme-linked immunosorbent assay (ELISA) and biolayer interferometry (BLI) methods were utilized for determining the binding potency of CR3022 with SARS-CoV-2 spike. The study of Tian et al. suggests that the epitope of CR3022 does not overlap with ACE2 but with the epitope of SARS-CoV-2 RBD [28]. However, another report by Wrapp et al. indicates the higher binding affinity of SARS-CoV-2 with ACE2 [29]. Human antibodies such as m396, 80R, S230, and F26G19 are docked well with SARS-CoV-2, whereas CR3022 exhibit good affinity with SARS-CoV-2 based on Rosetta docking [30]. The hydrophobic component of CR3022 as well as spike could be the reason for the interaction [31, 32]. Mab362 human antibody overlaps with ACE2 receptor and binds with SARS-CoV-2 and weakly with SARS-CoV-1 [33]. ASN440, SER375, ASN437, SER373, and ALA372 are the residues involved in binding of Wild with SER27E, ARG58, TYR27D, and TYR92 residues of antibody (Fig. 4a). The shortest hydrogen bond distance is 1.9 Å which is between ASN437 and TYR92. Table 3 shows the CLUSpro binding energy and interacting residues between the antibody and the spike protein. The binding energy of wild type is − 42.6 kcal/mol, and energies of other models lie between − 71 and − 100 kcal/mol. Model 2 shows the maximum interaction and least binding energy (− 99.7 kcal/mol). LYS528, ASN370, and PHE374 and ALA372 are the interacting amino acids of the spike in Model2_CR3022 (Fig. 4b). CR3022 neutralizing antibody has shown increased binding affinity with Wild and decreased binding affinity with Delta. Binding energy of other models is far away from the current energy. Therefore, this model is used for the molecular dynamics calculation.

**Table 3:** CLUSpro binding energy and interacting residues between antibody and spike protein

Models	CLUSpro energy	Interacting residues within 2.7 Å	
		SARS-CoV-2 SP	CR3022
**Model 1**	− 71.9	ASP427, GLN414, LYS378, TYR369	ASP54, TYR27, GLU56, LYS73
**Model 2**	− 99.7	LYS528, ASN370, PHE374, ALA372	TYR52, GLU56, TYR27D, TYR92, SER27E, SER27E, ASP54
**Model 3**	− 74.4	ASP427, GLN414, LYS378, TYR369	SER27F, TYR27D, GLU56, ASP54, LUS73
**Model 4**	− 74.7	ASP427, GLN414, LYS378, TYR369	SER27F, TYR27D, GLU56, ASP54, LYS73
**Model 5**	− 71.0	ASP427, GLN414, LYS378, TYR369	SER27F, TYR27D, GLU56, ASP54, LYS73
**Model 6**	− 72.9	LYS444, ARG452, GLU471	TYR92, TYR27D, SER99, ASP54, GLU56, LYS73, TRP47

### Molecular dynamics simulations

**Fig. 5:**
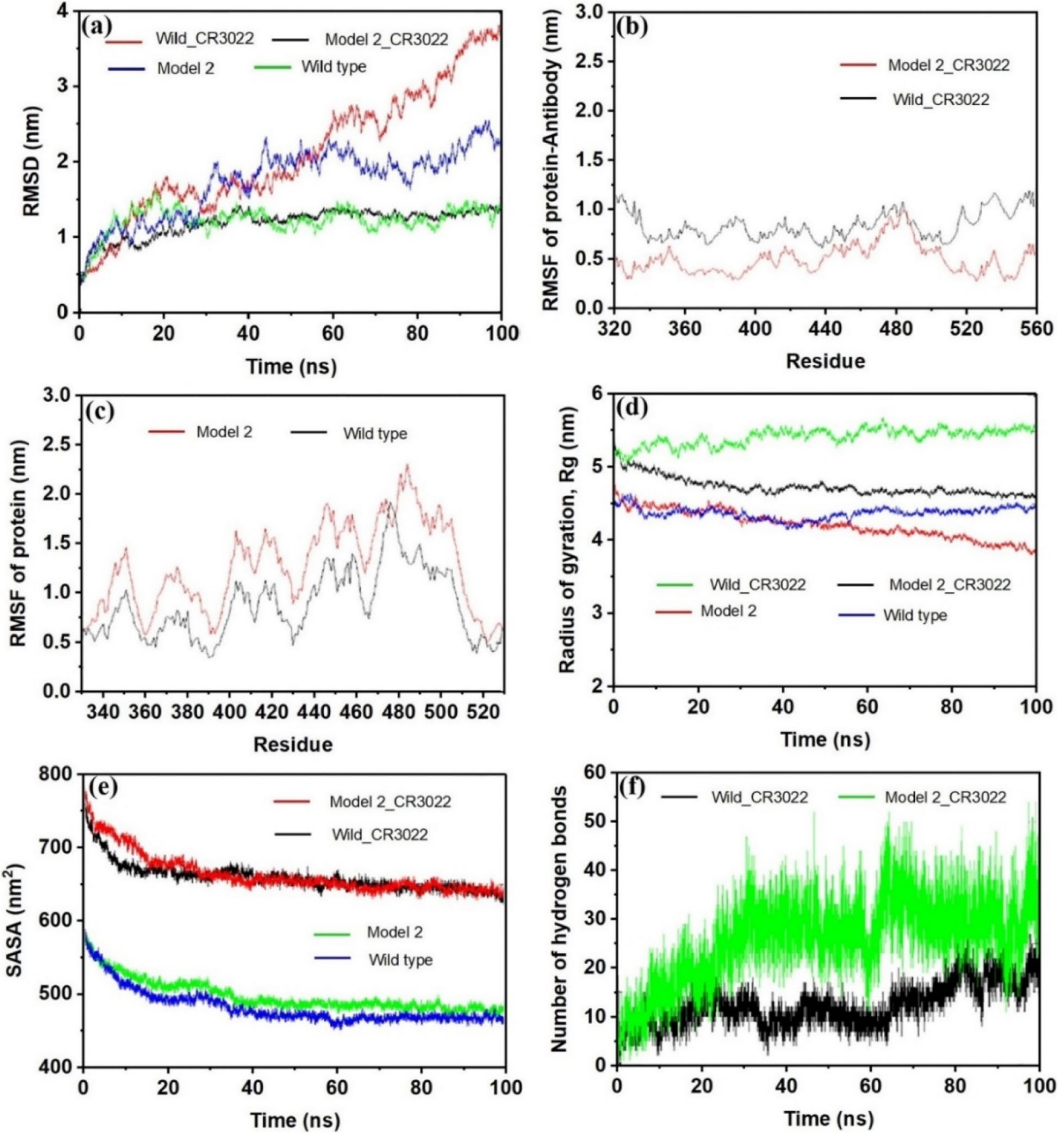
**a** The observed RMSD with respect to time. **b** Time versus RMSF of protein antibody for Wild_CR3022 and Model2_CR3022. **c** Time versus RMSF of protein antibody for Wild type and Model2. **d** Time versus radius of gyration. **e** SASA with respect to time. **f** Observed number of hydrogen bonds with respect to simulation time

E484K mutation on new variants shows repulsion to various antibodies which could be the reason for the significant resistance to the neutralization of vaccine sera [34]. Based on the docking results, we have taken Model2 with antibody for dynamics calculation in order to determine the stability of the complex. Figure 5a represents the RMSD values of proteins (Model2 and Wild) and its complexes (Model2_CR3022 and Wild_CR3022) for 100-ns simulation. Like other molecular dynamics simulations, initial run time of all proteins and its complexes exhibits flexibility in RMSD due to the amino acid changes.

As seen, Model2, Model2_CR3022, and the Wild type become stable within 20–30 ns, whereas Wild_CR3022 does not attain stability. In the case of Model2_CR3022, initially the RMSD value gradually increases until 1 nm, and thereafter no much increment is observed. Moreover, no significant variation is observed after RMSD reached ~ 1.3 nm at 40 ns. The Wild_CR3022 keeps gaining RMSD and requires more simulation run for stability confirmation. High variation is also observed in the case of Model2. In this case, after RMSD reaches 2 nm in 45 ns, no steady state can be observed. The Model2_CR3022 and Wild type exhibit nearly similar trend of RMS deviation after reaching stability. Yu et al. reported that CR3022 with SARS-CoV is more stable in comparison with CR3022 with SARS-CoV-2, and common conservative amino acids are TYR369, PHE377, LYS378, TYR380, GLY381, LYS386, and LEU390 [35]. Significant difference of RMSD between Model2 and Wild suggests that mutation makes a drastic change in the protein structure.

Root-mean-square fluctuation (RMSF) of protein/complex was also analyzed, and the profiles are provided in Fig. 5b and c. As seen, Model2 shows higher fluctuation as compared to Wild type; however, the overall fluctuation for each residue seems to be same meaning that no significant conformational change of residues occurs even after the mutation. Model2_CR3022 shows marginal fluctuations on RMSF which clearly indicates that the residue numbers 342 to 400 and residue numbers 510 to 532 are more flexible in RBD of protein. The flexibility of these residues in the mutated protein could be the reason for the strong interaction with antibody compared to the Wild type. The average RMSF of Wild, Model2, Wild_CR3022, and Model2_CR3022, respectively, correspond to values 0.9 nm, 1.7 nm, 0.4 nm, and 0.7 nm. The RMSD and RMSF analysis indicates higher stability of mutated spike complex in comparison with the Wild.

Stability analysis is also performed from the analysis of radius of gyration (Rg) of proteins and its complexes. Rg is another factor from which we can determine structural compactness, stability, and folding of proteins. As demonstrated by Fig. 5d, the Rg values correspond to Model2_CR3022, and Model2 decreases as simulation time increases. This indicates the highest compactness, good stability, and more folded nature of these structures [36]. The average value of Rg for Model2_CR3022 is 4.8 nm, and for Model2, it is 4.3 nm. The observed Rg value of Wild remains consistent until the end of the simulation. On the other hand, Wild_CR3022 exhibits a slight increment after finishing simulation, indicating a slight instability. The solvent accessible surface area (SASA) is used to understand the solvent accessibility of proteins [37]. Figure 5e clearly indicates that Model2_CR3022 and Wild_CR3022 have similar and higher SASA (650 nm^2^) than that of unbound proteins (490 nm^2^). These bound complexes show high solvation effect. Moreover, both the bound complexes are more open and diffused; hence, the amino acid residues are well exposed to the environment. Furthermore, the SASA values are decreasing slowly with simulation time which indicates conformational changes of residues. A large number of hydrogen bonds (an average of 30 hydrogen bonds) were observed in the Model2_CR3022 (Fig. 5f). A previous study claimed that SARS-CoV-2 forms a salt bridge at LYS417 with GLU329 of ACE2 [38]. In the present study, we observed that Model2 forms average of 17 hydrogen bonds with CR3022, whereas the Wild forms average of 12 hydrogen bonds with antibody. It is notable that 10–11 average hydrogen bonds between Wild with ACE2 were reported [39]. The greater number of hydrogen bonds between RDB and paratope of CR3022 in Model2_CR3022 shows maximum stability in comparison with Wild_CR3022 [40].

**Fig. 6:**
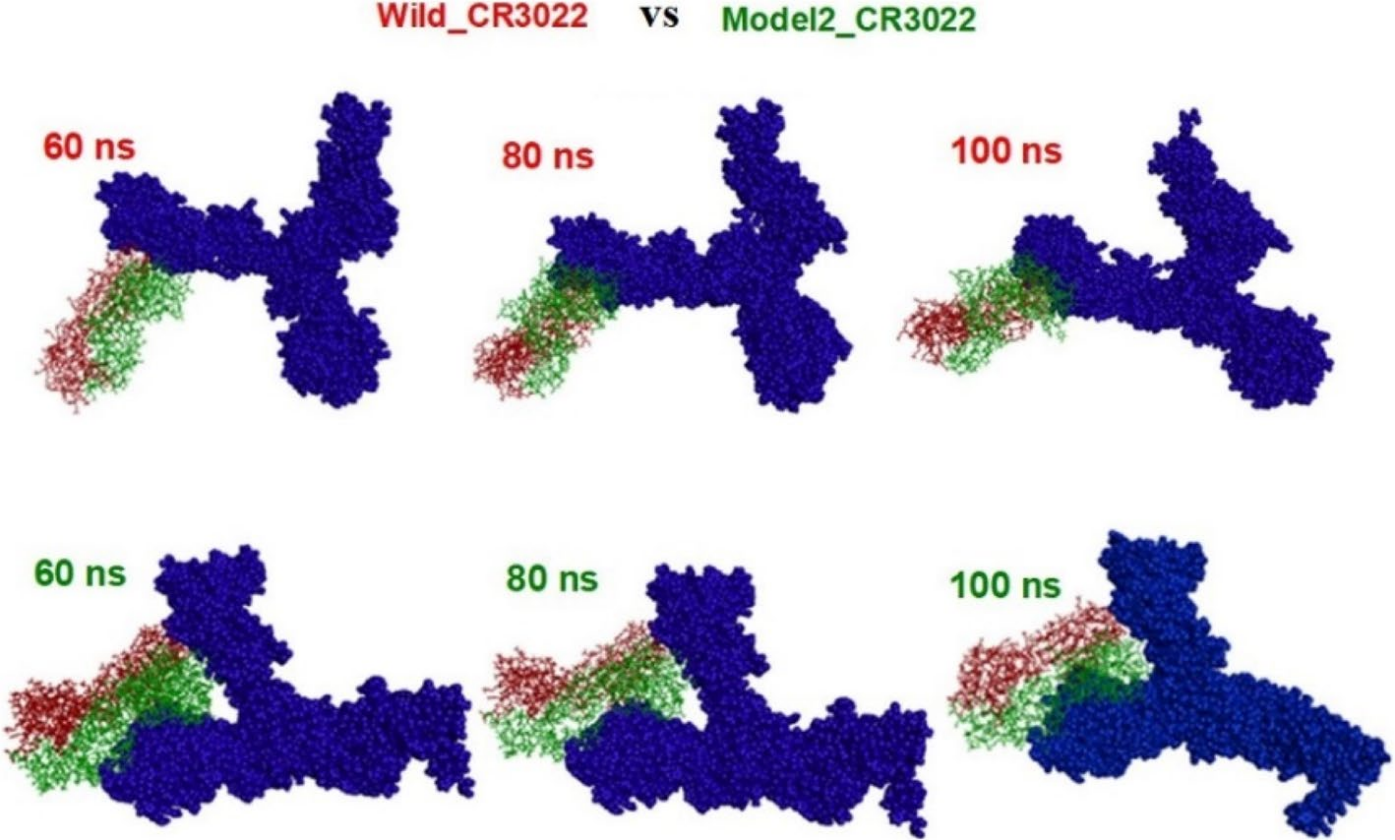
Snapshot of Wild_CR3022 and Model2_CR3022 at the time intervals, 60 nm, 80 ns, and 100 ns time of molecular dynamics simulation (top row, Wild_CR3022 and bottom row, Model2_CR3022)

Next, we explored the analysis of trajectory files of two systems in order to understand the overall appearance of the complex systems and mode of interactions of spikes with antibody. The snapshot structures at 60 ns, 80 ns, and 100 ns of these two systems, Wild_CR3022 and Model2_CR3022, are shown in Fig. 6. It is evident that Wild shows one side interaction with CR3022, whereas Delta interacts in such a way that CR3022 is placed in between the two branches of spike. This gives extra stability for Model2_CR3022, and hence, one can see numerous interactions during the simulation times. Interactions involved in Model2 are GLY252, ASP253, GLN23, ARG21, ASN137, GLN134, ASP111, THR109, VAL83, ASN87, LYS535, ASN532, LYS528, LEU368, CYS379, PHE377, ALA372, ASN370, and SER371. The interacting residues of Wild type are GLN506, ASN437, PRO499, ASN439, SER438, ASN440, ASN343, SER371, SER373, and GLN506. These interacting residues are taken from the last frame of the trajectory file after MD simulation.

### MM/GBSA analysis

The results summarized in Table 4 present total binding free energy and its four components. Reported MM/GBSA value of SARS-CoV-2 Wild with ACE is − 60.8 kcal/mole [41], whereas in the current study the observed free energy for SARS-CoV-2 Wild with CR3022 is − 103.31. This indicates the higher binding affinity of Wild with CR3022 than ACE2. A significant contribution to energy minimization is mainly from van der Waals energy and electrostatic energy, whereas desolvation energy (generalized born model) increases the total energy. The calculated binding energy indicates the higher affinity for mutated spike protein as compared to the Wild type which is consistent with results from docking and molecular dynamics simulations. Decomposition analysis of each residue of mutated spike is also done, and the results (supporting info, Fig. S5) reveal that ASP253 residue shows a maximum van der Waals energy of − 6.64 kcal/mol, electrostatic energy of − 58.12 kcal/mol, born desolvation energy of 60.15 kcal/mol, and surface area of − 1.11 kcal/mol. Van der Waals energy of Model2_CR3022 is twice as that Wild_CR3022, and similar trend was also found for electrostatic contribution. This clearly indicates the higher flexibility of antibody to the mutated spike in comparison with Wild type. Furthermore, the van der Waals interaction is almost similar to electrostatic energy, which indicates that alkyl and benzyl moieties of mutated spike will not show significant interaction with antibody.

**Table 4:** Total binding free energy and its components of Model2_CR3022 and Wild_CR3022

**Parameters**	**Model2_CR3022** (kcal/mol)	**Wild_CR3022** (kcal/mol)
Van der Waals energy	− 262.64	− 134.52
Electrostatic energy	− 277.45	− 174.56
Generalized born model	431.86	222.46
Surface area	− 32.20	− 16.68
Total binding free energy	− 140.42	− 103.31

### Analysis of interactions of CR3022 and Delta with six vaccines

For docking studies, we employed six launched/marketed vaccines, namely, etesevimab, bebtelovimab, BD-368–2, imdevimab, bamlanivimab, and casirivimab. Table 5 indicates the observed CLUSpro energy of these antibodies with Wild type and Delta, and docked complexes are seen in Fig. S4. In all these cases, the binding affinity of Delta with antibodies decreases significantly (− 61.7 to − 112.0 kcal/mol). In comparison with Delta, we can see higher binding affinity (− 165.1 to − 298.3 kcal/mol) with docked systems for the Wild type. Substantial loss of neutralizing activity due to change in sequence of spike was reported for these vaccines, which raises concern about the effectiveness of antibodies [42]. Upon comparing all vaccines, LYCoV016 show maximum interaction with Wild and Delta spikes. This vaccine is efficacious for neutralizing SARS-CoV-2 Wild type but not for Kappa [43].

**Table 5:** Docking score (kcal/mol) of Delta and Wild with six reported vaccine/antibodies

**Antibodies**	**PDB ID**	Wild (*kcal/mol)*	Delta* (kcal/mol)*
BD-CoV-1404	**7CHH**	− 230.0	− 65.3
Bebtelovimab	**7MMO**	− 279.9	− 93.6
REGN10933	**6XDG**	− 258.1	− 100.0
REGN10987	**6XDG**	− 165.1	− 61.7
LY-CoV016	**7C01**	− 298.3	− 112.0
Bamlanivimab	**7MKG**	− 292.4	− 79.8

**Fig. 7:**
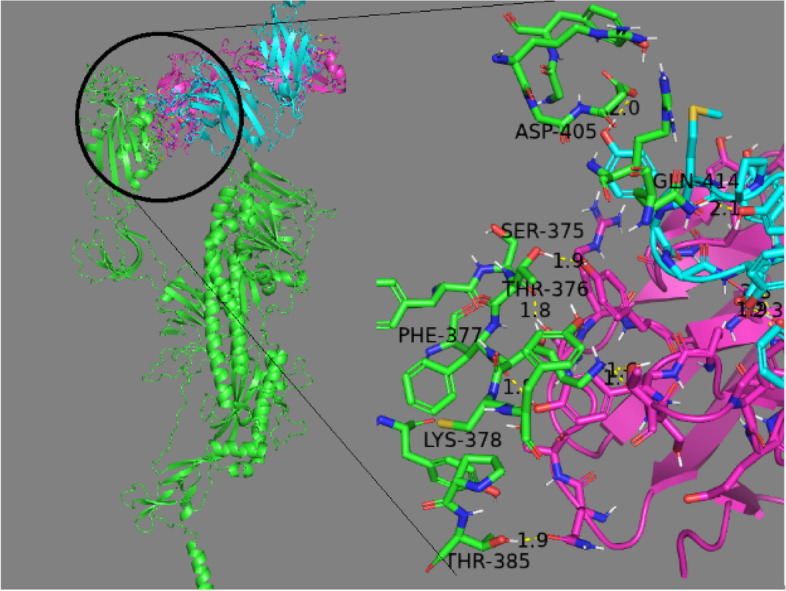
Docked system of LYCoV016 with Delta. The section highlighted by circle is enlarged and provided in the right side of the figure

The crucial residues SER375, THR376, PHE377, LYS378, THR385, ASP405, and GLN414 in RBD of Delta (Fig. 7) form a binding for LYCoV016, whereas ALA372, SER373, SER375, ASN437, and asn440 in RBD of Wild form a hydrogen bonding for LYCoV016. Owing to the mutations, Delta appears to show an increase in hydrogen bonds with these vaccines in comparison with Wild; nonetheless, there is a decrease in the CLUSpro score. Previously, Takuya et al. reported that vaccines such as REGN10987 and REGN10933 lost neutralization activity against B.1.351 and mink cluster 5 [44]. In all cases, antibodies failed to resist Delta, but they are significantly resistive to Wild spike. Importantly, our study suggests that mutated spikes or new variants may escape from the available antibodies since mutations can deter active residues of antibodies. Moreover, mutations may change the polarity or bulkiness nature of residues which ultimately leads to the structural change of protein and reduce probability of maximum interaction with CR3022 or any antibodies. Hence, more antibodies are developed, modified, or designed in order to find maximum interaction with spike or mutated spikes. Another concern is that mutation on the spike can trigger or reduce its interaction with ACE2. Searching a greater number of antibodies is important, and in-depth study in this regard is under process.

**Fig. 8:**
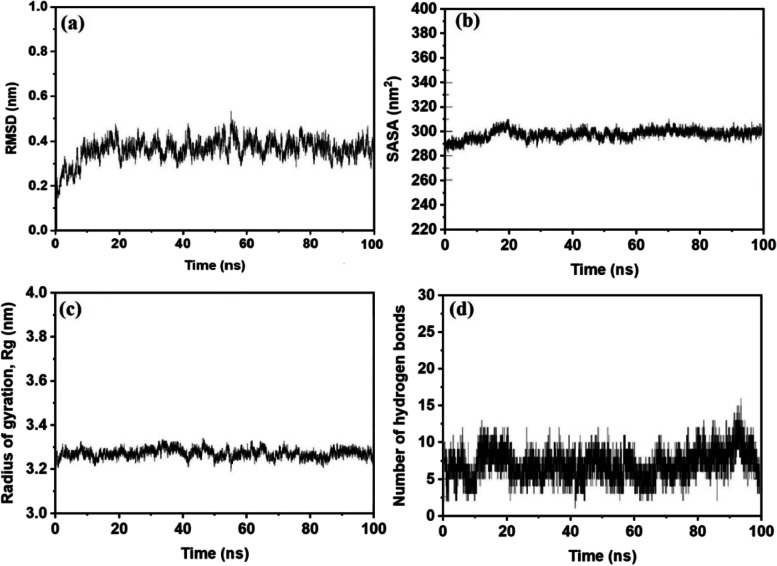
Results of 100-ns molecular dynamics simulation corresponding to LYCoV016. Order of figures. **a** RMSD profile, **b** SASA profile, **c** radius of gyration (Rg), and **d** number of hydrogen bond of M_LYCoV016 versus time

The molecular dynamics simulation (100 ns) is performed for the best-docked system (LYCoV016) in order to understand the stability, flexibility, solvent-accessible area, and compactness of proteins (Fig. 8a, b, c, and d: RMSD, SASA, Rg, and number of hydrogen bonds). It is observed that the RMSD exhibit lower fluctuations (< 0.5) and stabilize after 10 ns, whereas the radius of gyration reveals the better compactness and the folding nature. Hydrogen bonds are the main deciding factor for the stability of overall complexes. The present system exhibits 8 hydrogen bonds in average throughout the simulation. SASA seems to be high and decreasing over simulation time. This clearly indicates that the residues are well exposed to the environment. From the MD results, it is understandable that LYCoV016_Delta exhibits good stability.

## Conclusions

This study reveals that SARS-CoV-2 Delta possesses high homology similarity towards Wild and its interactions with human antibody. The CR3022 antibody exhibits high binding affinity (− 99.7 kcal/mol). The best-docked system of Model2 with Delta shows better MMPBSA binding free energy as compared to Wild type. Moreover, folding free energy and mutations increases significantly for Delta in comparison with Wild type. Notably, the studied six antibodies show decreased antigen resistance with Delta. The results reveal that docked structures of six antibodies with Wild type exhibit numerous interactions, whereas substantially lowered interactions with Delta can be witnessed. The present study highlights the necessity to develop new antibodies which should be applicable even for other variants. Investigation with more antibodies is in progress for further strengthening these assertions.

## Supplementary Information

**Additional file 1:** **Fig. S1.** Predicted local similarity to target, Normalised QMEAN4 score, Ramachandran pot and predicted models. **Fig. S2.** Sequence of all models obtained from PSIPRED online server. **Fig. S3.** ∆G (kcal/mol) versus Temperature (degree) obtained from Scoop server for (A)Model1 (B) Model2 (C) Model3(D) Model4 (E) Model5 (F) Model5 (G) Wild type. **Fig. S4.** Best docked systems of Wild and Model2 with various antibodies. **Fig. S5.** VDW, ELE, SA and GB contributions of RBD Delta with respect to residue numbers. **Table S1.** ΔΔG (kcal/mol) and ΔΔS ENCoM and ΔΔG DynaMut of Model 1. **Table S2.** ΔΔG (kcal/mol) and ΔΔS ENCoM and ΔΔG DynaMut of Model 3. **Table S3.** ΔΔG (kcal/mol) and ΔΔS ENCoM and ΔΔG DynaMut of Model 4. **Table S4.** ΔΔG (kcal/mol) and ΔΔS ENCoM and ΔΔG DynaMut of Model 5. **Table S5.** ΔΔG (kcal/mol) and ΔΔS ENCoM and ΔΔG DynaMut of Model 6. **Table S6.** ΔΔG (kcal/mol) of Model 1 predicted by mCSM, SDM and DUET method. **Table S7.** ΔΔG (kcal/mol) of Model 2 predicted by mCSM, SDM and DUET method. **Table S8.** ΔΔG (kcal/mol) of Model 3 predicted by mCSM, SDM and DUET method. **Table S9.** ΔΔG (kcal/mol) of Model 4 predicted by mCSM, SDM and DUET method. **Table S10.** ΔΔG (kcal/mol) of Model 5 predicted by mCSM, SDM and DUET method. **Table S11.** ΔΔG (kcal/mol) of Model 6 predicted by mCSM, SDM and DUET method. **Table S12.** ∆H_m,_ ∆C_p_,T_m_ and ∆G_r_ obtained from Scoop online server.

## Data Availability

In accordance with journal principles, sharing of all simulation input files and sequence data are available on reasonable request.
