# Supplementary material for: Insights on the interaction of SARS-CoV-2 variant B.1.617.2 with antibody CR3022 and analysis of antibody resistance
Source: J Genet Eng Biotechnol. 2023 Mar 20;21:35. doi: 10.1186/s43141-023-00492-y (PMC10026237; doi:10.1186/s43141-023-00492-y)
Supplement: Supplementary file 1 — Additional file 1: Fig. S1. Predicted local similarity to target, Normalised QMEAN4 score, Ramachandran pot and predicted models. Fig. S2. Sequence of all models obtained from PSIPRED online server. Fig. S3. ∆G (kcal/mol) versus Temperature (degree) obtained from Scoop server for (A)Model1 (B) Model2 (C) Model3(D) Model4 (E) Model5 (F) Model5 (G) Wild type. Fig. S4. Best docked systems of Wild and Model2 with various antibodies. Fig. S5. VDW, ELE, SA and GB contributions of RBD Delta with respect to residue numbers. Table S1. ΔΔG (kcal/mol) and ΔΔS ENCoM and ΔΔG DynaMut of Model 1. Table S2. ΔΔG (kcal/mol) and ΔΔS ENCoM and ΔΔG DynaMut of Model 3. Table S3. ΔΔG (kcal/mol) and ΔΔS ENCoM and ΔΔG DynaMut of Model 4. Table S4. ΔΔG (kcal/mol) and ΔΔS ENCoM and ΔΔG DynaMut of Model 5. Table S5. ΔΔG (kcal/mol) and ΔΔS ENCoM and ΔΔG DynaMut of Model 6. Table S6. ΔΔG (kcal/mol) of Model 1 predicted by mCSM, SDM and DUET method. Table S7. ΔΔG (kcal/mol) of Model 2 predicted by mCSM, SDM and DUET method. Table S8. ΔΔG (kcal/mol) of Model 3 predicted by mCSM, SDM and DUET method. Table S9. ΔΔG (kcal/mol) of Model 4 predicted by mCSM, SDM and DUET method. Table S10. ΔΔG (kcal/mol) of Model 5 predicted by mCSM, SDM and DUET method. Table S11. ΔΔG (kcal/mol) of Model 6 predicted by mCSM, SDM and DUET method. Table S12. ∆Hm, ∆Cp,Tm and ∆Gr obtained from Scoop online server. [file 43141_2023_492_MOESM1_ESM.docx]

Insights on the interaction of SARS-CoV-2 variant B.1.617.2 with antibody CR3022 and analysis of antibody resistance

**Supporting information (Tables and Figures)**

Figure S1. Predicted local similarity to target, Normalised QMEAN4 score, Ramachandran pot and predicted models. 7

Figure S2. Sequence of all models obtained from PSIPRED online server. 13

Figure S3. ∆G (kcal/mol) versus Temperature (degree) obtained from Scoop server for (A)Model1 (B) Model2 (C) Model3(D) Model4 (E) Model5 (F) Model5 (G) Wild type. 13

Figure S4. Best docked systems of Wild and Model2 with various antibodies. 15

Figure S5. VDW, ELE, SA and GB contributions of RBD Delta with respect to residue numbers. 17

Table S1. ΔΔG (kcal/mol) and ΔΔS ENCoM and ΔΔG DynaMut of Model 1 7

Table S2. ΔΔG (kcal/mol) and ΔΔS ENCoM and ΔΔG DynaMut of Model 3 7

Table S3. ΔΔG (kcal/mol) and ΔΔS ENCoM and ΔΔG DynaMut of Model 4 7

Table S4. ΔΔG (kcal/mol) and ΔΔS ENCoM and ΔΔG DynaMut of Model 5 9

Table S5. ΔΔG (kcal/mol) and ΔΔS ENCoM and ΔΔG DynaMut of Model 6 9

Table S6. ΔΔG (kcal/mol) of Model 1 predicted by mCSM, SDM and DUET method. 9

Table S7. ΔΔG (kcal/mol) of Model 2 predicted by mCSM, SDM and DUET method. 10

Table S8. ΔΔG (kcal/mol) of Model 3 predicted by mCSM, SDM and DUET method. 10

Table S9. ΔΔG (kcal/mol) of Model 4 predicted by mCSM, SDM and DUET method. 11

Table S10. ΔΔG (kcal/mol) of Model 5 predicted by mCSM, SDM and DUET method. 11

Table S11. ΔΔG (kcal/mol) of Model 6 predicted by mCSM, SDM and DUET method. 11

Table S12. ∆H_m,_ ∆C_p_ ,T_m_ and ∆G_r_ obtained from Scoop online server. 12

Model1


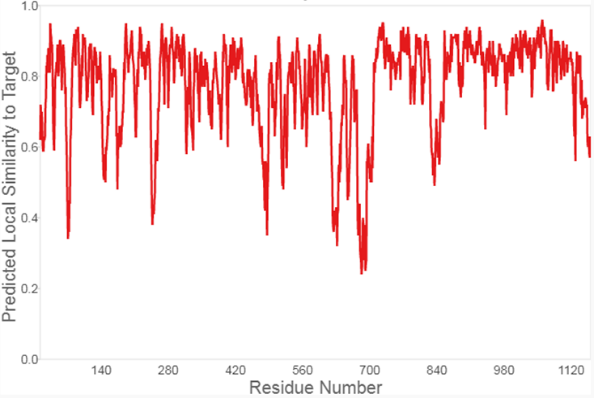

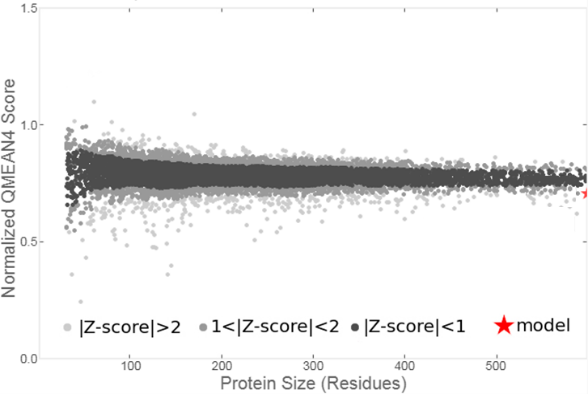


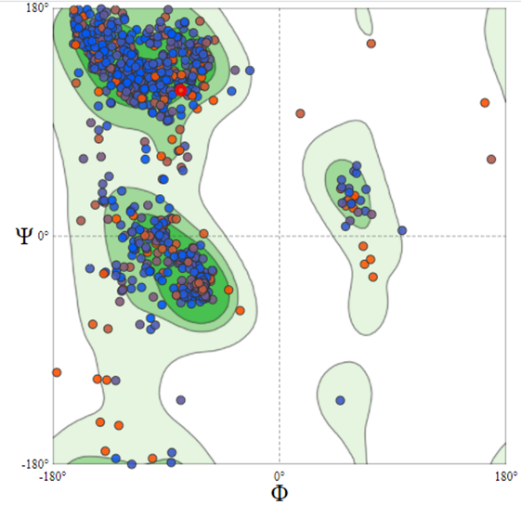

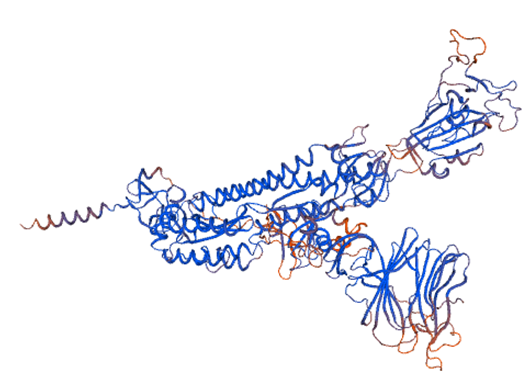


Model 2


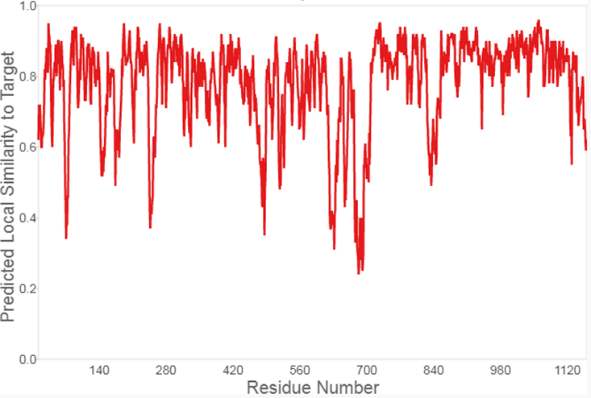

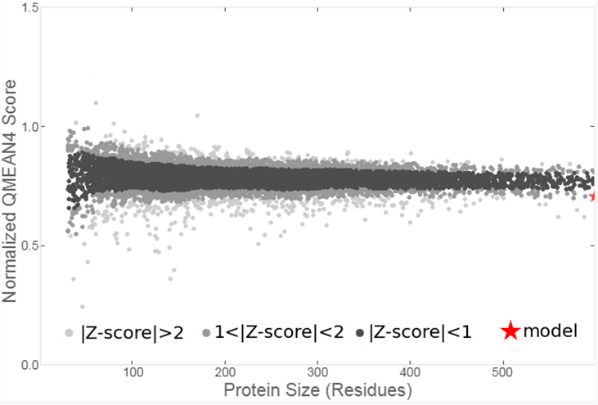


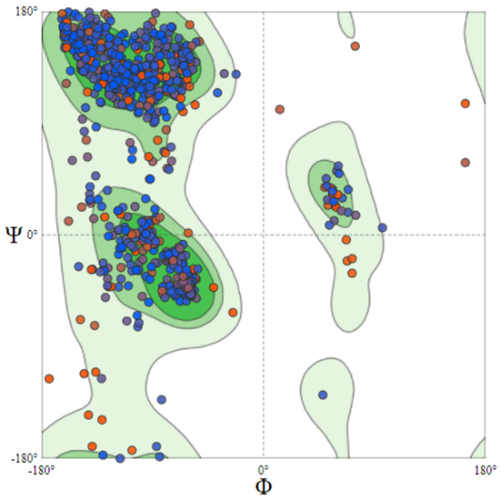

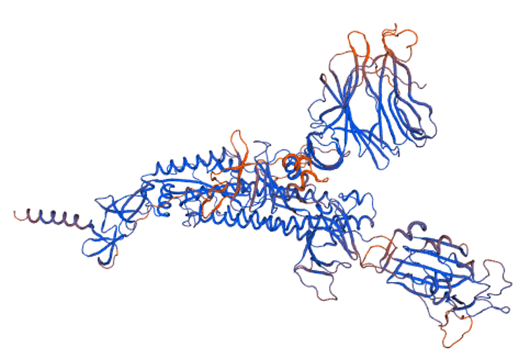


Model 3


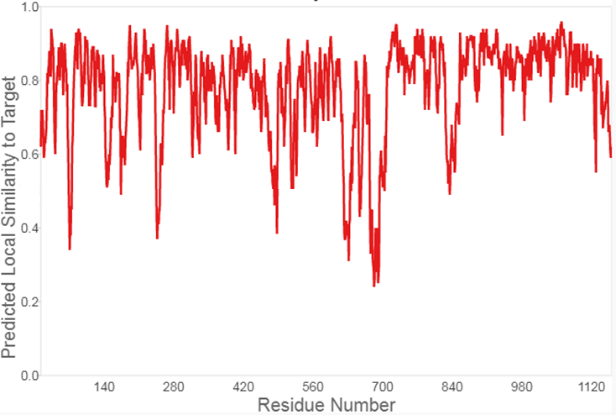

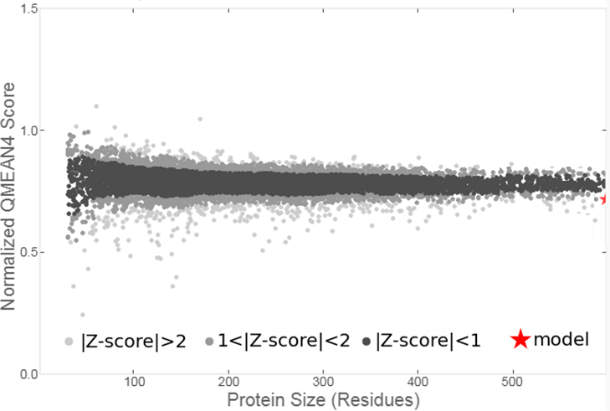


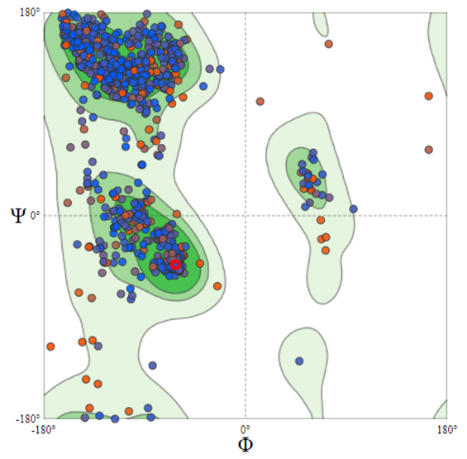

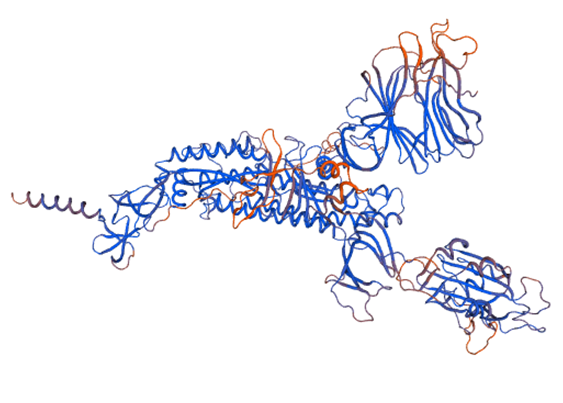


Model4


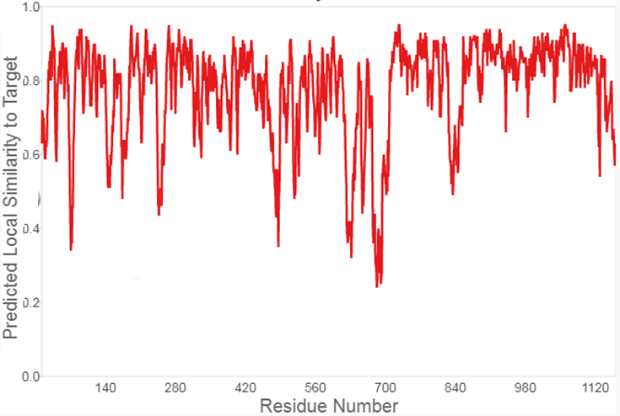

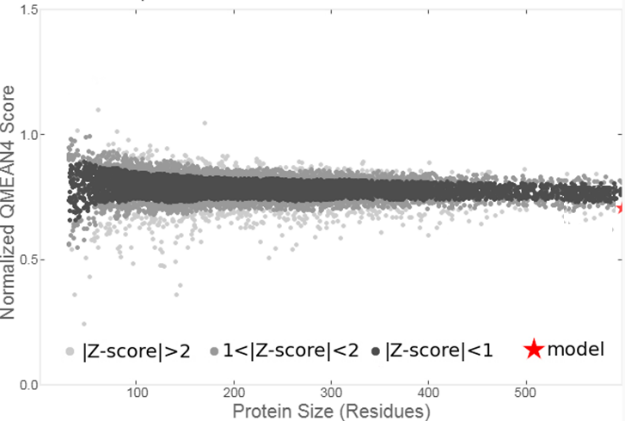


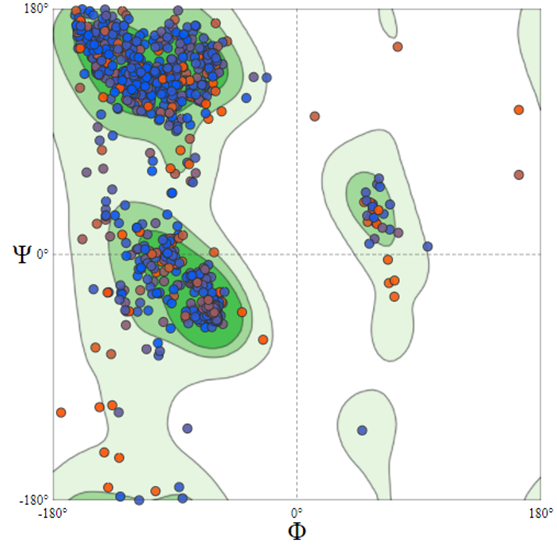

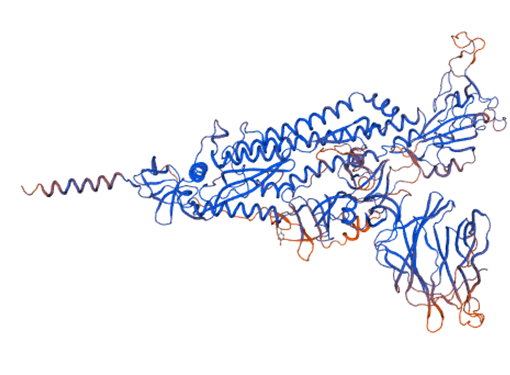


Model5


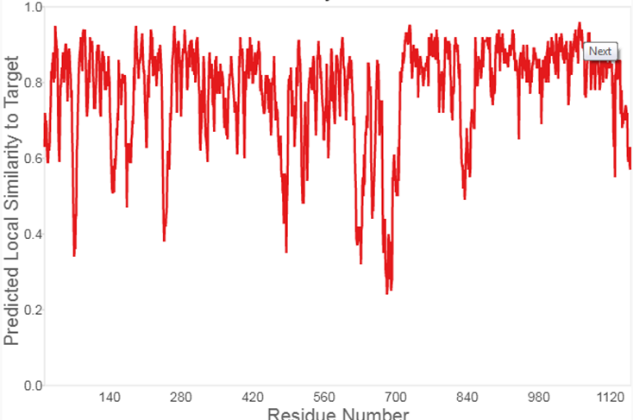

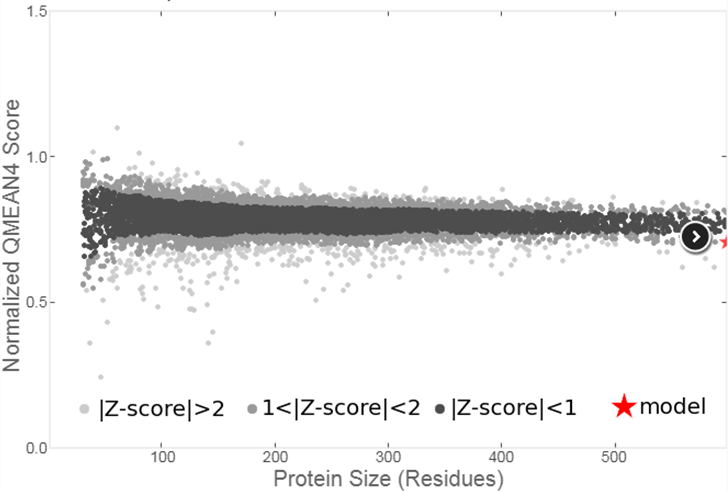


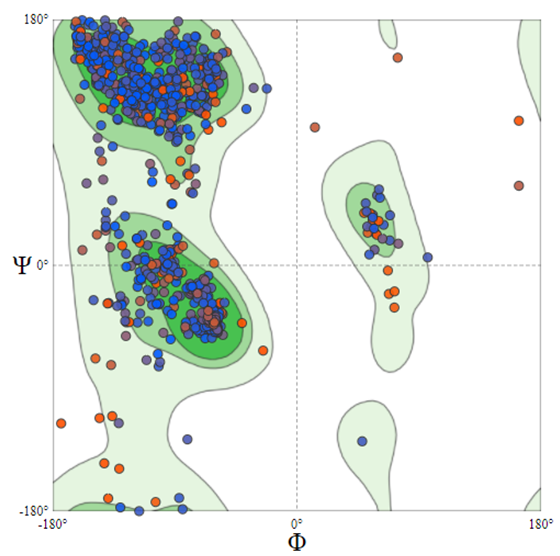

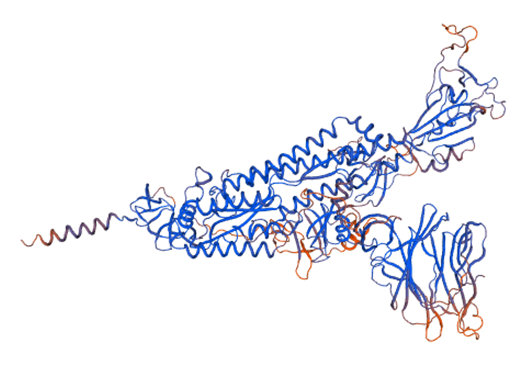


Model 6


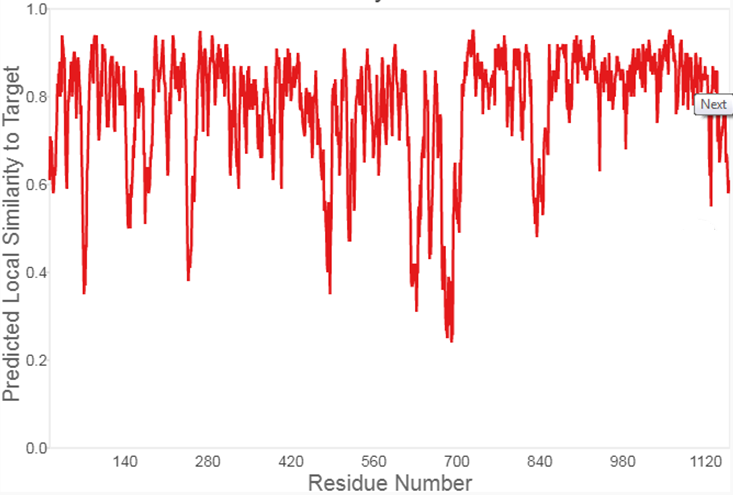

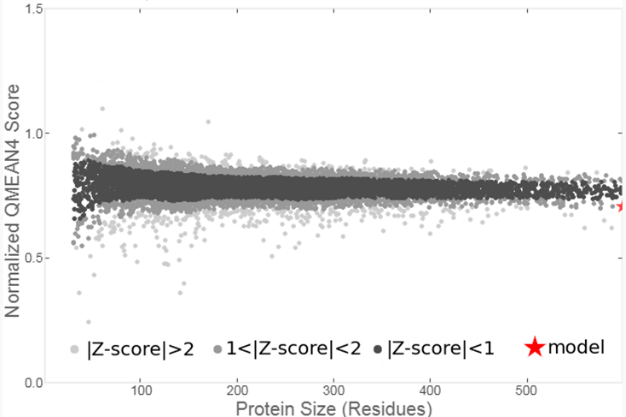


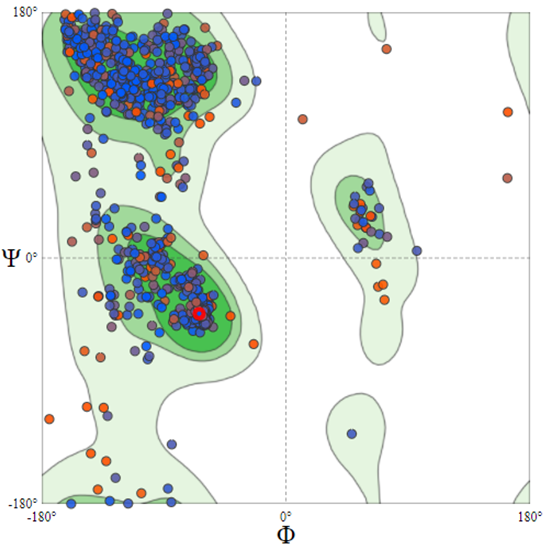

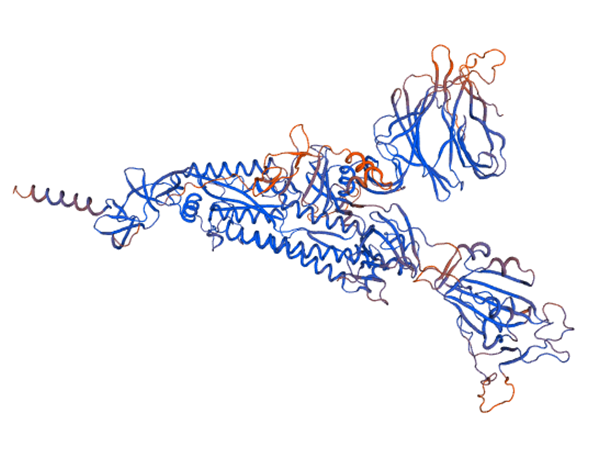


Figure S1. Predicted local similarity to target, Normalised QMEAN4 score, Ramachandran pot and predicted models.
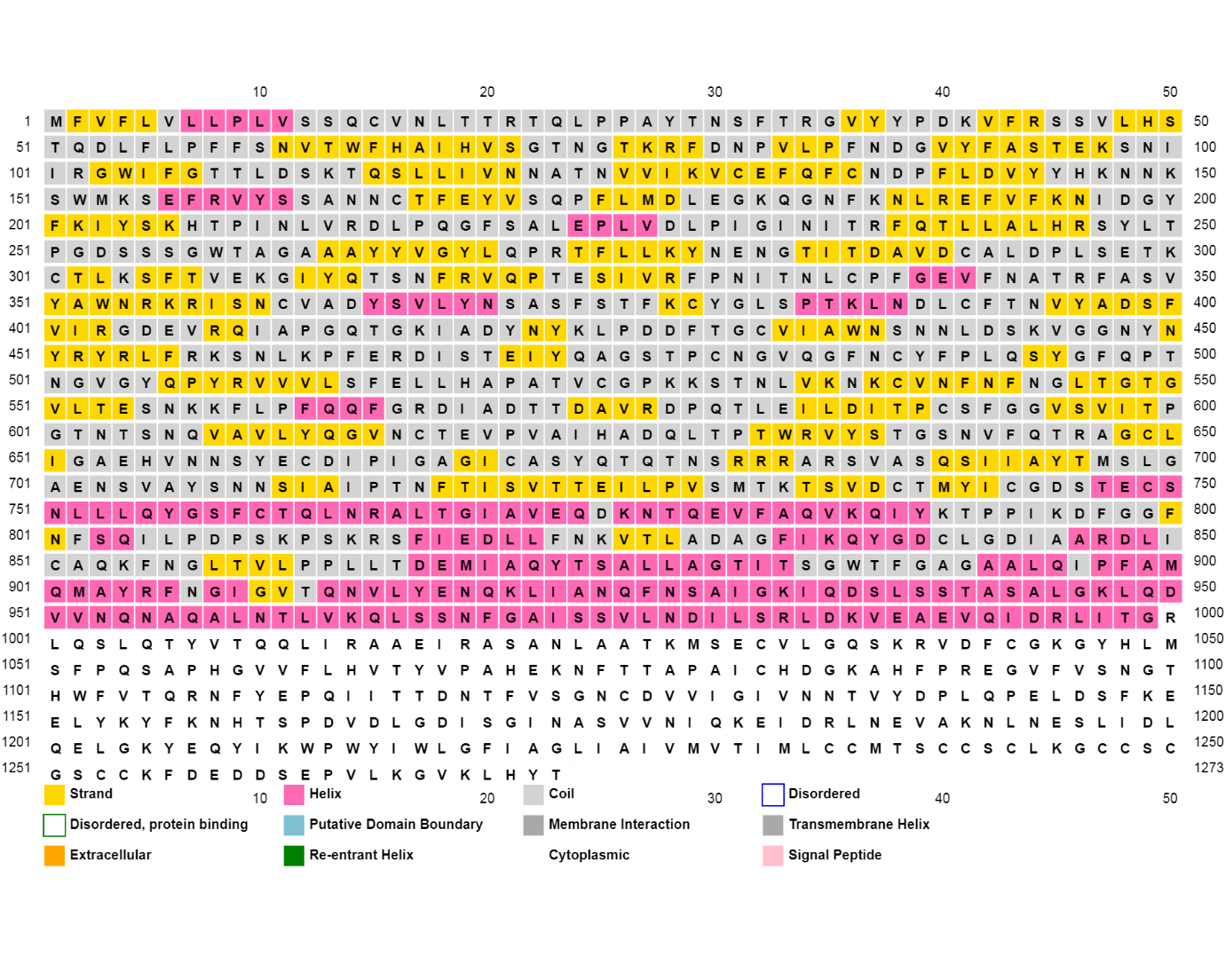


| Serial no. | AA from | AA to | Position | ΔΔG ENCoM (kcal/mol) | ΔΔS ENCoM(kcal/mol) | ΔΔG DynaMut (kcal/mol) |
| --- | --- | --- | --- | --- | --- | --- |
| 1 | L | R | 452 | 0.142 | -0.178 | 0.511 |
| 2 | Q | H | 1071 | 0.003 | -0.004 | 0.138 |
| 3 | E | Q | 484 | 0.07 | -0.088 | 0.439 |
| 4 | D | G | 614 | -0.106 | 0.133 | 0.548 |
| 5 | P | R | 681 | 0.373 | -0.467 | 0.467 |

Table S1. ΔΔG (kcal/mol) and ΔΔS ENCoM and ΔΔG DynaMut of Model 1

| Serial no. | AA from | AA to | Position | ΔΔG ENCoM(kcal/mol) | ΔΔS ENCoM (kcal/mol) | ΔΔG DynaMut (kcal/mol) |
| --- | --- | --- | --- | --- | --- | --- |
| 1 | G | D | 142 | 0.431 | -0.539 | 1.105 |
| 2 | T | I | 95 | 0.231 | -0.289 | 1.689 |
| 3 | L | R | 452 | 0.142 | -0.178 | 0.511 |
| 4 | Q | H | 1071 | 0.003 | -0.004 | 0.138 |
| 5 | E | Q | 484 | 0.07 | -0.088 | 0.439 |
| 6 | D | G | 614 | -0.106 | 0.133 | 0.548 |

Table S2. ΔΔG (kcal/mol) and ΔΔS ENCoM and ΔΔG DynaMut of Model 3

| Serial no. | AA from | AA to | Position | ΔΔG ENCoM (kcal/mol) | ΔΔS ENCoM (kcal/mol) | ΔΔG DynaMut (kcal/mol) |
| --- | --- | --- | --- | --- | --- | --- |
| 1 | G | D | 142 | 0.431 | -0.539 | 1.105 |
| 2 | E | K | 154 | 0.022 | -0.027 | 0.757 |
| 3 | L | R | 452 | 0.142 | -0.178 | 0.511 |
| 4 | V | L | 382 | 0.256 | -0.32 | 0.398 |
| 5 | E | Q | 484 | 0.07 | -0.088 | 0.439 |
| 6 | D | G | 614 | -0.106 | 0.133 | 0.548 |

Table S3. ΔΔG (kcal/mol) and ΔΔS ENCoM and ΔΔG DynaMut of Model 4

| Serial no. | AA from | AA to | Position | ΔΔG ENCoM(kcal/mol) | ΔΔS ENCoM(kcal/mol) | ΔΔG DynaMut(kcal/mol) |
| --- | --- | --- | --- | --- | --- | --- |
| 1 | G | D | 142 | 0.431 | -0.539 | 1.105 |
| 2 | E | K | 154 | 0.022 | -0.027 | 0.757 |
| 3 | L | R | 452 | 0.142 | -0.178 | 0.511 |
| 4 | E | Q | 484 | 0.07 | -0.088 | 0.439 |
| 5 | D | G | 614 | -0.106 | 0.133 | 0.548 |

Table S4. ΔΔG (kcal/mol) and ΔΔS ENCoM and ΔΔG DynaMut of Model 5

| Serial no. | AA from | AA to | Position | ΔΔG ENCoM  (kcal/mol) | ΔΔS ENCoM  (kcal/mol) | ΔΔG DynaMut  (kcal/mol) |
| --- | --- | --- | --- | --- | --- | --- |
| 1 | G | D | 142 | 0.431 | -0.539 | 1.105 |
| 2 | L | R | 452 | 0.142 | -0.178 | 0.511 |
| 3 | D | G | 614 | -0.106 | 0.133 | 0.548 |

Table S5. ΔΔG (kcal/mol) and ΔΔS ENCoM and ΔΔG DynaMut of Model 6

| Residues | ΔΔG mCSM | ΔΔG SDM | ΔΔG DUET |
| --- | --- | --- | --- |
| 452 | -1.052 | -0.460 | -0.703 |
| 1071 | -0.461 | 0.470 | -0.110 |
| 484 | -0.144 | -0.690 | 0.021 |
| 614 | -0.291 | 2.330 | 0.399 |
| 681 | 0.193 | -0.030 | 0.387 |

Table S6. ΔΔG (kcal/mol) of Model 1 predicted by mCSM, SDM and DUET method.

| Residues | ΔΔG mCSM | ΔΔG SDM | ΔΔG DUET |
| --- | --- | --- | --- |
| 142 | -1.130 | -1.280 | -1.044 |
| 154 | 0.537 | -0.250 | 0.820 |
| 452 | -1.052 | -0.460 | -0.703 |
| 382 | -0.509 | -1.250 | -0.479 |
| 1071 | -0.461 | 0.470 | -0.110 |
| 484 | -0.144 | -0.690 | 0.021 |
| 614 | -0.291 | 2.330 | 0.399 |
| 681 | 0.193 | -0.030 | 0.387 |

Table S7. ΔΔG (kcal/mol) of Model 2 predicted by mCSM, SDM and DUET method.

| Residues | ΔΔG mCSM | ΔΔG SDM | ΔΔG DUET |
| --- | --- | --- | --- |
| 142 | -1.130 | -1.280 | -1.044 |
| 95 | -0.560 | 1.910 | 0.219 |
| 452 | -1.052 | -0.460 | -0.703 |
| 1071 | -0.461 | 0.470 | -0.110 |
| 484 | -0.144 | -0.690 | 0.021 |
| 614 | -0.291 | 2.330 | 0.399 |

Table S8. ΔΔG (kcal/mol) of Model 3 predicted by mCSM, SDM and DUET method.

| Residues | ΔΔG mCSM | ΔΔG SDM | ΔΔG DUET |
| --- | --- | --- | --- |
| 142 | -1.130 | -1.280 | -1.044 |
| 154 | 0.537 | -0.250 | 0.820 |
| 452 | -1.052 | -0.460 | -0.703 |
| 382 | -0.509 | -1.250 | -0.479 |
| 484 | -0.144 | -0.690 | 0.021 |
| 614 | -0.291 | 2.330 | 0.399 |

Table S9. ΔΔG (kcal/mol) of Model 4 predicted by mCSM, SDM and DUET method.

| Residues | ΔΔG mCSM | ΔΔG SDM | ΔΔG DUET |
| --- | --- | --- | --- |
| 142 | -1.130 | -1.280 | -1.044 |
| 154 | -1.044 | -0.250 | 0.820 |
| 452 | -1.052 | -0.460 | -0.703 |
| 484 | -0.144 | -0.690 | 0.021 |
| 614 | -0.291 | 2.330 | 0.399 |

Table S10. ΔΔG (kcal/mol) of Model 5 predicted by mCSM, SDM and DUET method.

| Residues | ΔΔG mCSM | ΔΔG SDM | ΔΔG DUET |
| --- | --- | --- | --- |
| 142 | -1.130 | -1.280 | -1.044 |
| 452 | -1.052 | -0.460 | -0.703 |
| 614 | -0.291 | 2.330 | 0.399 |

Table S11. ΔΔG (kcal/mol) of Model 6 predicted by mCSM, SDM and DUET method.

|  |  |  |  |  |
| --- | --- | --- | --- | --- |
|  |  |  |  |  |
|  |  |  |  |  |
|  |  |  |  |  |
|  |  |  |  |  |
|  |  |  |  |  |
|  |  |  |  |  |
|  |  |  |  |  |
|  |  |  |  |  |
|  |  |  |  |  |
|  |  |  |  |  |
|  |  |  |  |  |
|  |  |  |  |  |
|  |  |  |  |  |
|  |  |  |  |  |
|  |  |  |  |  |
|  |  |  |  |  |
|  |  |  |  |  |
|  |  |  |  |  |
|  |  |  |  |  |
|  |  |  |  |  |
|  |  |  |  |  |
|  |  |  |  |  |
|  |  |  |  |  |

| Models | ∆H_m_ | ∆C_p_ | T_m_ | ∆G_r_ |
| --- | --- | --- | --- | --- |
| Wild | -2.6 | -0.06 | 78.8 | -0.1 |
| Model1 | -100.7 | -2.41 | 77.3 | -5.1 |
| Model2 | -107.9 | -2.62 | 76.3 | -5.5 |
| Model3 | -108.9 | -2.67 | 75.4 | -5.5 |
| Model 4 | -100.5 | -2.38 | 77.8 | -5.1 |
| Model 5 | -98.7 | -2.35 | 77.8 | -5.1 |
| Model 6 | -109.8 | -2.73 | 74.5 | -5.5 |

Table S12. ∆H_m,_ ∆C_p_ ,T_m_ and ∆G_r_ obtained from Scoop online server.


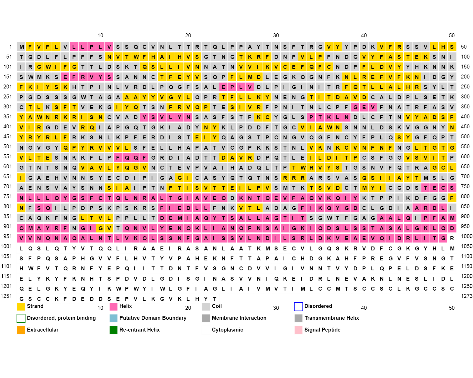

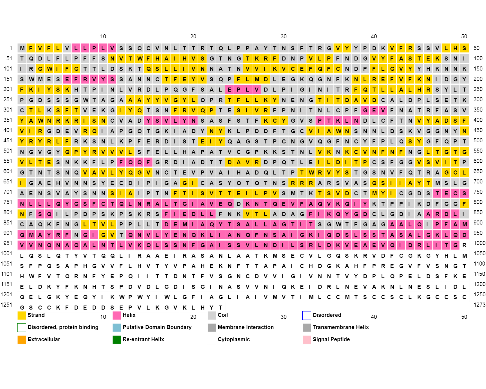


Model 1 Model2


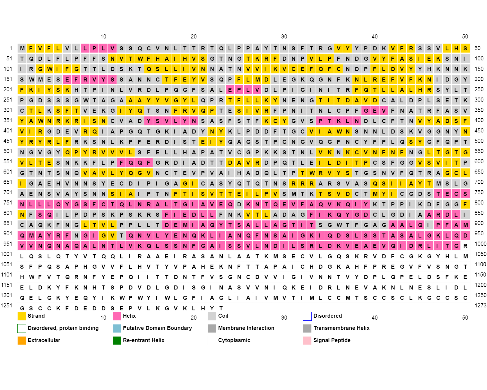

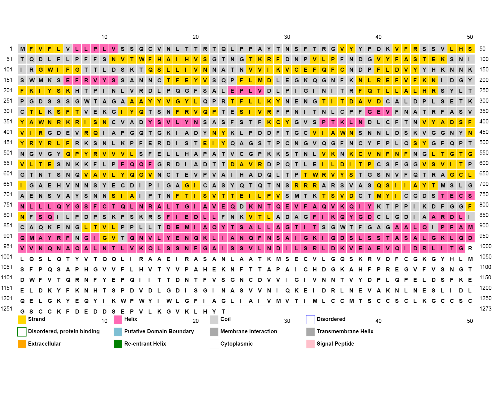


Model 3 Model 4


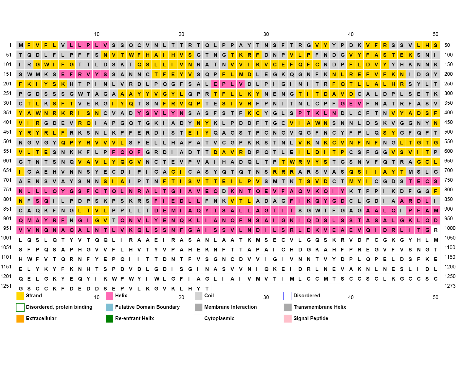

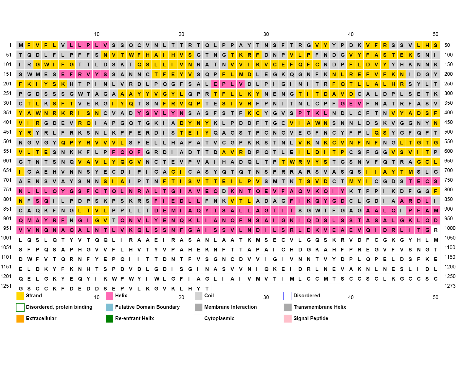


Model 5 Model 6


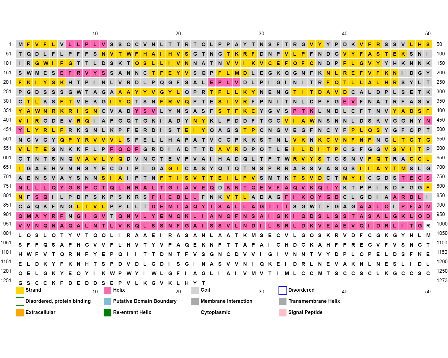


Wild

Figure S2. Sequence of all models obtained from PSIPRED online server.


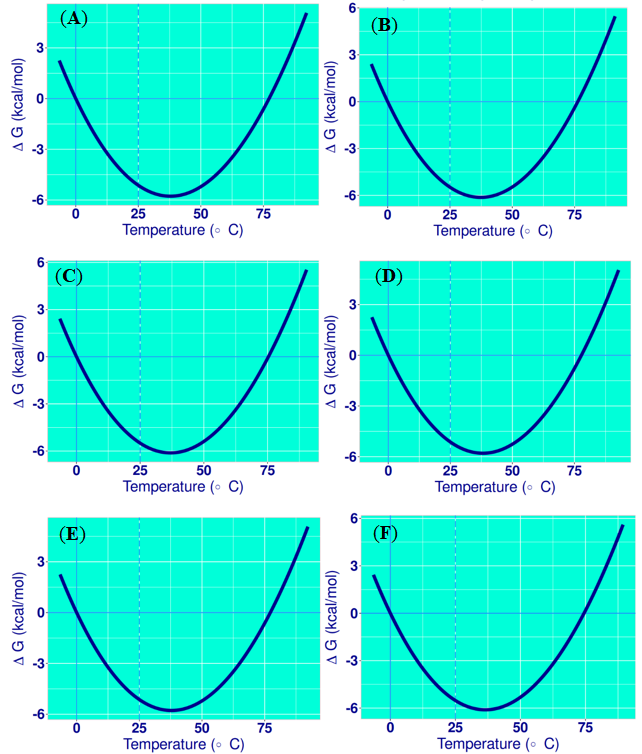

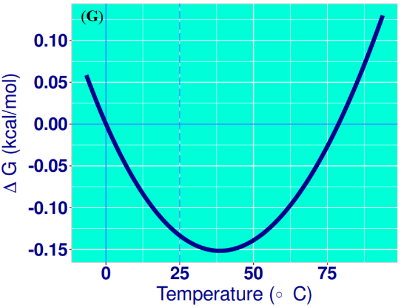


Figure S3. ∆G (kcal/mol) versus Temperature (degree) obtained from Scoop server for (A) Model1 (B) Model2 (C) Model3(D) Model4 (E) Model5 (F) Model5 (G) Wild.


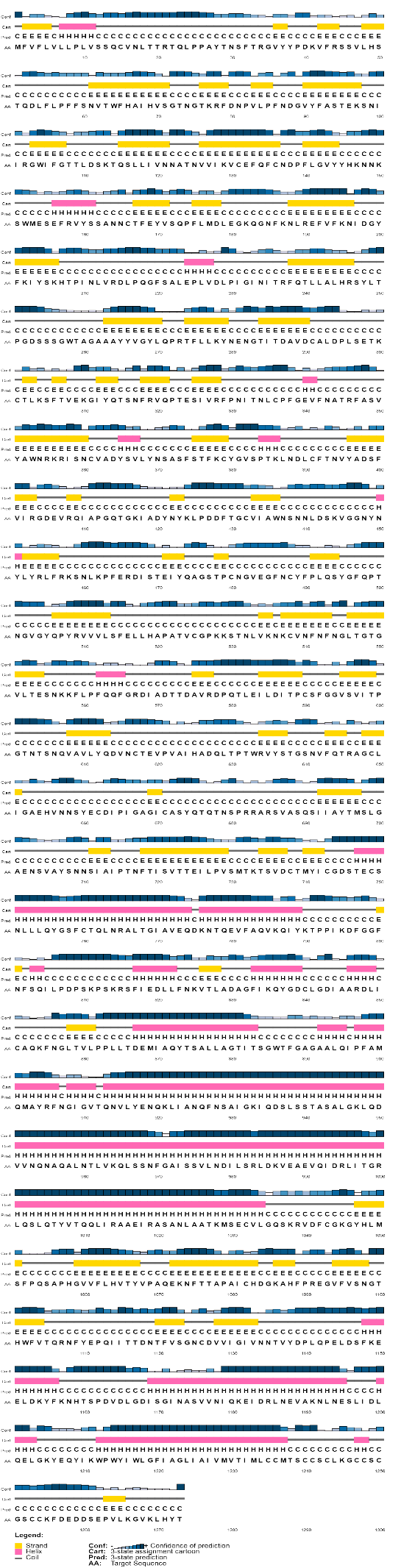

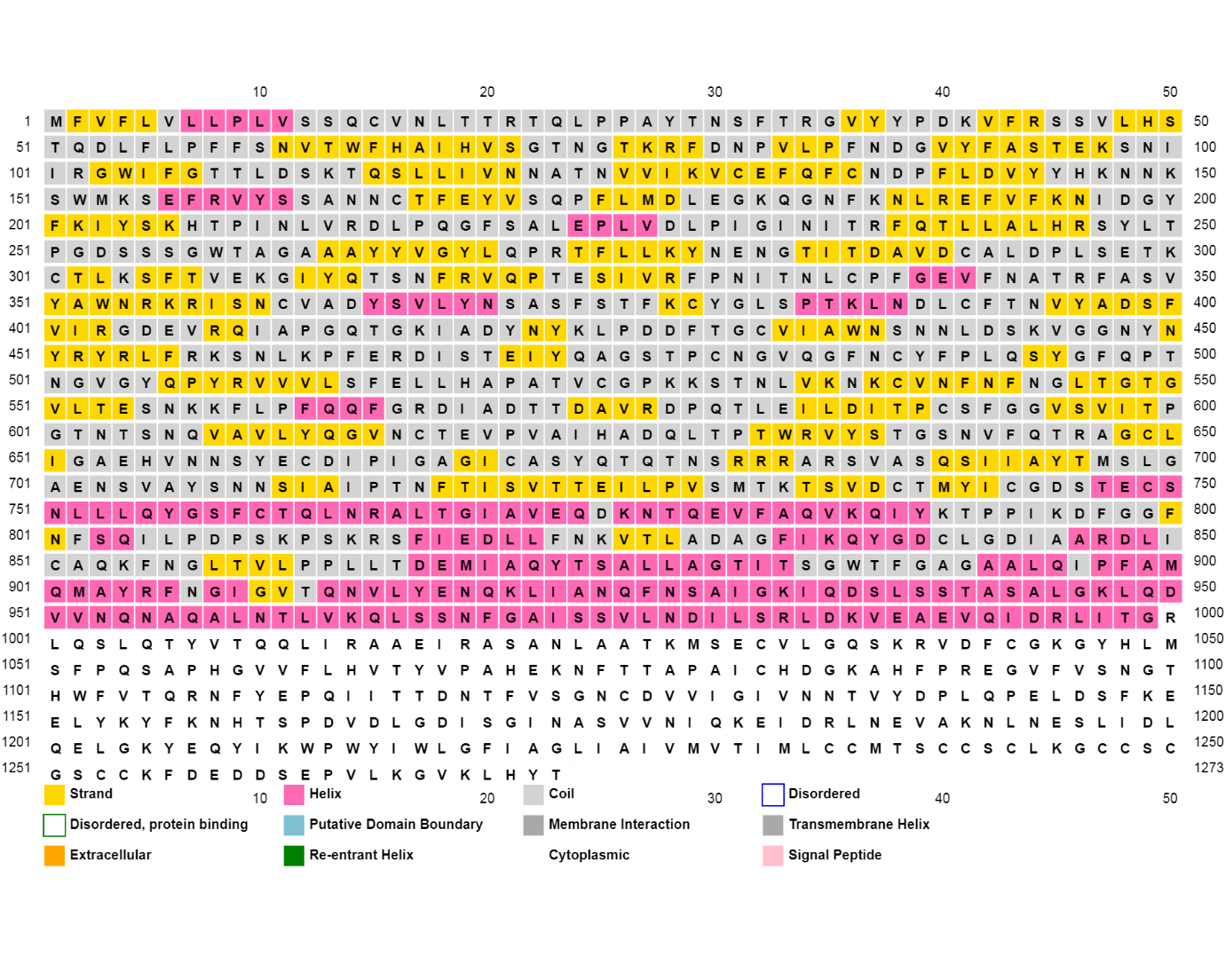


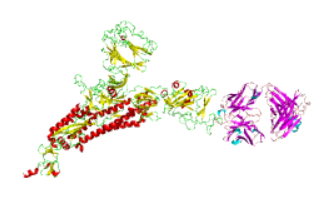

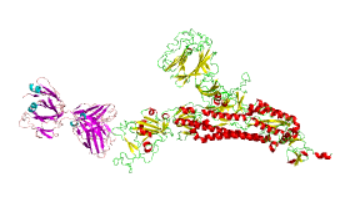


Wild_7mkg Wild_6xdgB


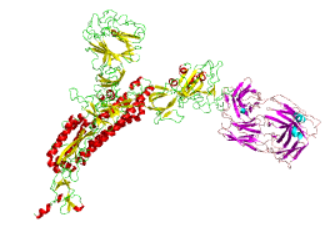

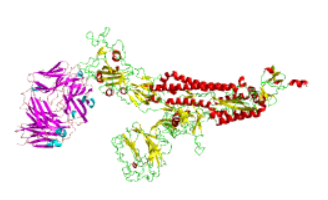


Wild_6xdgA Wild_7mmo


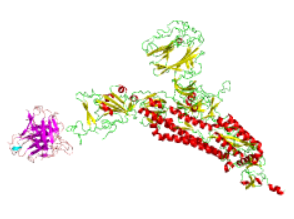

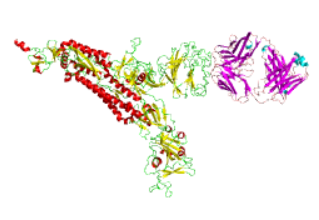


Wild_7chh Wild_7c01


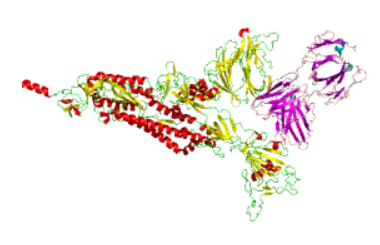

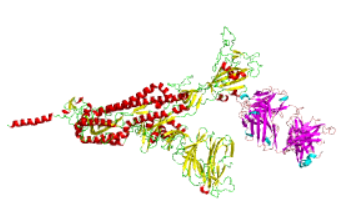


### Model2_6xdgA Model2_7mkg


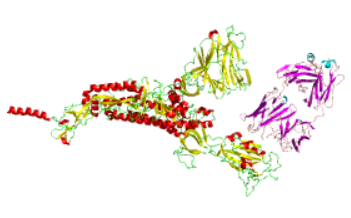

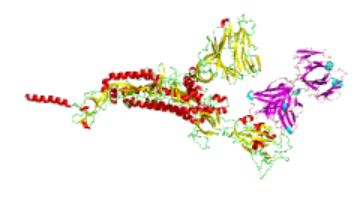


### Model2_6xdgB Model2_7mmo


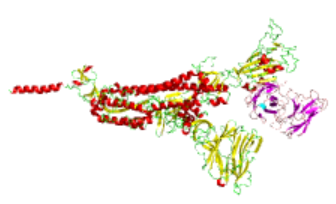

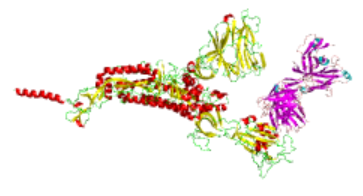


### Model2_7chh Model2_7c01

Figure S4. Best docked systems of Wild and Model2 with various antibodies.

Figure S5. VDW, ELE, SA and GB contributions of RBD Delta with respect to residue numbers.
